# Realization of efficient quantum gates with a superconducting qubit-qutrit circuit

**DOI:** 10.1038/s41598-019-49657-1

**Published:** 2019-09-16

**Authors:** T. Bækkegaard, L. B. Kristensen, N. J. S. Loft, C. K. Andersen, D. Petrosyan, N. T. Zinner

**Affiliations:** 1 Department of Physics and Astronomy, Aarhus University, DK-8000 Aarhus C, Denmark; 2 Department of Physics, ETH Zürich, CH-8093 Zürich, Switzerland; 3 Institute of Electronic Structure and Laser, FORTH, GR-71110 Heraklion, Greece; 4 Aarhus Institute of Advanced Studies, Aarhus University, DK-8000 Aarhus C, Denmark

**Keywords:** Superconducting devices, Quantum information, Theoretical physics

## Abstract

Building a quantum computer is a daunting challenge since it requires good control but also good isolation from the environment to minimize decoherence. It is therefore important to realize quantum gates efficiently, using as few operations as possible, to reduce the amount of required control and operation time and thus improve the quantum state coherence. Here we propose a superconducting circuit for implementing a tunable system consisting of a qutrit coupled to two qubits. This system can efficiently accomplish various quantum information tasks, including generation of entanglement of the two qubits and conditional three-qubit quantum gates, such as the Toffoli and Fredkin gates. Furthermore, the system realizes a conditional geometric gate which may be used for holonomic (non-adiabatic) quantum computing. The efficiency, robustness and universality of the presented circuit makes it a promising candidate to serve as a building block for larger networks capable of performing involved quantum computational tasks.

## Introduction

Richard Feynman famously suggested to simulate quantum physics with quantum computers^1^. Fourteen years later, Seth Lloyd proved that an array of spins with tunable interactions indeed represents a universal quantum simulator^2^. Dynamically controlled spin chains can realize analog quantum simulations and digital quantum computations. Several physical systems are being explored for implementing tunable spin chains in the quantum regime, including trapped ions and atoms^3–5^, quantum dots^6^ and superconducting circuits^7,8^. Over the last decade, superconducting circuits have steadily improved to become one of the most prominent candidates for the realization of scalable quantum computing^9–12^. With the development of the transmon qubit^13^ and further advances, such as the 3D transmon^10^, coherence times above 44 μs^14–17^ and per-step multi-qubit gate fidelity at the fault tolerance threshold for surface code error correction^18^ have been achieved on multi-qubit devices^19^.

Many approaches for entangling quantum gates with superconducting qubits have been implemented experimentally^19–27^ and many more have been proposed theoretically^28–33^. Still, as the search for better coherence, lower error rates and faster quantum gate operation times continues, more efficient universal realizations of key operations for a quantum processor are needed. Most implementations so far have used only one- and two-qubit quantum operations for realizing important multi-qubit gates^34^ such as the three-qubit quantum Toffoli^35^ (ccnot) and Fredkin^36^ (cswap) gates, requiring a theoretical minimum of five two-qubit gates^34,37,38^. This large number of required gates can be remedied by the use of a higher-lying state of a qutrit which can simplify the implementation of e.g. the Toffoli gate to three two-qubit gates as implemented optically in^39^ and in superconducting circuits in^40^. Moreover, the current implementations are highly specialized, meaning that the fabricated superconducting circuit is used just to implement a single three-qubit gate. A single circuit implementing several important universal quantum gates with high fidelity and minimal external control is therefore desirable.

Here, we propose a superconducting circuit realizing two qubits and a qutrit in between. First we show how the circuit can be used to generate two- and three-qubit entangled states^34,41,42^. Then we discuss how to implement the Fredkin gate using only two three-qubit operations, and the Toffoli gate using two (one-qubit) Hadamard gates and a three-qubit gate employing the intrinsic Ising-like *ZZ* (longitudinal) couplings and an external one-qubit driving. Finally we discuss how to implement a double-controlled universal unitary single-qubit gate. To illustrate this capability, we use the *ZZ*-couplings to realize the double-controlled holonomic gate^43,44^. The geometric nature of the holonomic gate provides robustness, but the first proposals required adiabatic control leading to more time for errors to occur. The increase in errors was partly avoided by the introduction of decoherence-free subspaces^45^, which can significantly reduce the detrimental effects of noise. We will instead implement a non-adiabatic generalization^46^, circumventing many of these difficulties. Furthermore, we will show that this double-controlled gate can be used to implement the three-qubit Deutsch gate and is therefore universal for quantum computing in itself, adding yet another tool in our toolbox for efficient quantum gates.

## Results

### Effective Hamiltonian of the system

**Figure 1:**
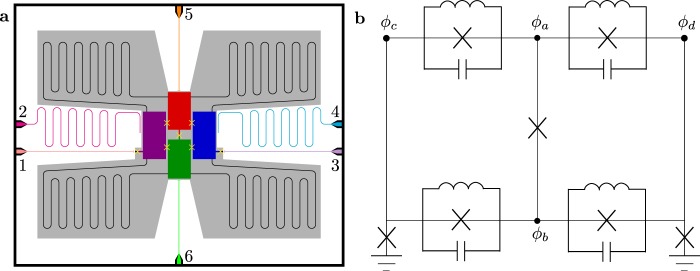
(**a**) Sketch of a possible physical implementation of the proposed circuit. Each colored box is a superconducting island corresponding to a node in a lumped circuit element model. Josephson junctions are shown schematically as yellow crosses. Bent black wires are inductors. The numbered colored lines are controls for readout and driving of the circuit: 1 and 3 are the flux lines for frequency tuning of the outer qubits, 2 and 4 are resonators capacitively coupled to left and right qubits, and lines 5 and 6 are control and driving of the two middle islands forming the middle qutrit. (**b**) Effective Lumped circuit scheme of the same circuit. The four nodes in the system are shown as dots and Josephson Junctions are shown as crosses.

Consider the circuit with four connected superconducting islands and the corresponding effective lumped circuit element model in Fig. 1. The top-bottom symmetry between the capacitors and the Josephson junctions cancels the direct exchange interaction mediated by the capacitances and the leading order term from the Josephson junctions. On the other hand, we choose an asymmetry in the inductive couplings such that the difference between them controls the Heisenberg-like exchange (transverse) coupling, which can be made arbitrarily small if so desired. Furthermore, while the exchange interaction mediated by the leading-order Josephson term (${E}_{J1}/2$ in Supplemental Note 1) is canceled by symmetry, the dispersive (*ZZ*) coupling survives. The couplings are defined in Eq. (A61) and as seen in Supplementary Note 2, a *ZZ* coupling strength of ~20 MHz is realistic. Moreover, the *ZZ* coupling can be tuned *in*-*situ* by an external flux.

**Figure 2:**
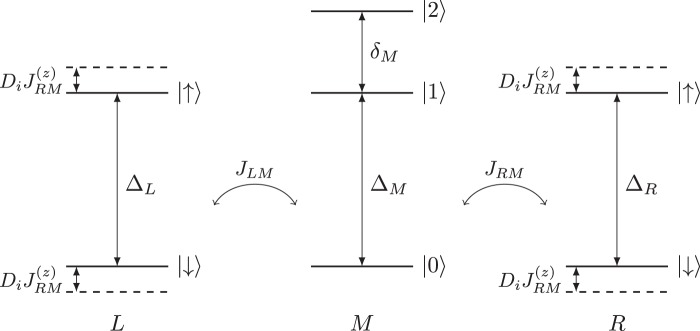
Energy diagram of the system of two qubits (left, *L*, and right, *R*) and a qutrit (middle, *M*) described by the Hamiltonian in Equation (1). Also shown are the exchange couplings *J*_*αM*_ and state-dependent energy shifts ${J}_{\alpha M}^{(z)}$ of (1). *D*_*i*_ depend on the state of the qutrit $|i\rangle$, with, ${D}_{0}=0$, and typically, ${D}_{1}\gtrsim 2$ and ${D}_{2}\lesssim 4$.

Using the effective lumped-element circuit of Fig. 1b and following the standard procedure for circuit quantization^13,47^, we derive the Hamiltonian of the system involving a suitable set of variable for the relevant dipole modes of the circuit, as detailed in the Method and in more details in the Supplementary Note 1. The Hamiltonian of the resulting qubit-qutrit-qubit system shown in Fig. 2 takes the form:

1 $$\begin{array}{rcl}H & = & \frac{1}{2}{\Delta }_{L}{\sigma }_{L}^{z}+{\Delta }_{M}|1\rangle \langle 1|+({\Delta }_{M}+{\delta }_{M})\,|2\rangle \langle 2|+\frac{1}{2}{\Delta }_{R}{\sigma }_{R}^{z}\\  &  & +\,{J}_{L{M}_{01}}({\sigma }_{L}^{-}|1\rangle \langle 0|+{\sigma }_{L}^{+}|0\rangle \langle 1|)\\  &  & +\,{J}_{R{M}_{01}}({\sigma }_{R}^{-}|1\rangle \langle 0|+{\sigma }_{R}^{+}|0\rangle \langle 1|)\\  &  & +\,{J}_{L{M}_{12}}({\sigma }_{L}^{-}|2\rangle \langle 1|+{\sigma }_{L}^{+}|1\rangle \langle 2|)\\  &  & +\,{J}_{R{M}_{12}}({\sigma }_{R}^{-}|2\rangle \langle 1|+{\sigma }_{R}^{+}|1\rangle \langle 2|)\\  &  & +\,{J}_{LM}^{(z)}{\sigma }_{L}^{z}({D}_{1}|1\rangle \langle 1|+{D}_{2}|2\rangle \langle 2|)\\  &  & +\,{J}_{RM}^{(z)}{\sigma }_{R}^{z}({D}_{1}|1\rangle \langle 1|+{D}_{2}|2\rangle \langle 2|),\end{array}$$

where ${\sigma }_{\alpha }^{+}$ and ${\sigma }_{\alpha }^{-}$ are the spin-1/2 raising and lowering operators for the left (*α* = *L*) and right (*α* = *R*) qubits, ${\sigma }_{\alpha }^{z}$ is the Pauli *Z* operator, and Δ_*L*,*R*_ is the energy differences between the spin-up and spin-down states of the corresponding qubit. The states of the qutrit are denoted by $|j\rangle$ ($j=0,1,2$), Δ_*M*_ is the energy of state $|1\rangle$ and ${\Delta }_{M}+{\delta }_{M}$ is the energy of state $|2\rangle$, making the anharmonicity equal to ${\Delta }_{M}-{\delta }_{M}$, with the energy of the ground state $|0\rangle$ set to zero. Δ_*M*_ and *δ*_*M*_ can be tuned dynamically by an external flux if additional flux lines are added to the circuit, or by an AC-Stark shift stemming from off-resonant microwave driving^9^ using the lines 5 and 6 in Fig. 1(a) (see Supplementary Note 1). We note that the AC-Stark shift is included here for additional dynamical tuning of the circuit but it is not essential for the gate implementations discussed below.

The exchange (*XY*) coupling strengths between the qubits and the qutrit are given by *J*_*αM*_. Typically, the coupling ${J}_{\alpha {M}_{12}}$ ($\alpha =L,R$) to the $|1\rangle \leftrightarrow |2\rangle$ transition is stronger than the coupling ${J}_{\alpha {M}_{01}}$ to the $|0\rangle \leftrightarrow |1\rangle$ transition by a factor ~$\sqrt{2}$. The coefficients ${J}_{\alpha M}^{(z)}$ determine the dispersive (*ZZ*) interaction between the qubits and the qutrit with ${D}_{1}\gtrsim 2$ and ${D}_{2}\lesssim 4$, with the two parameters converging at 3 as the mixing is increased via the off-resonant driving. For clarity, these parameters are shown in Fig. 2. In a perfectly left-right symmetric circuit, we have ${\Delta }_{L}={\Delta }_{R}$ and ${J}_{LM}={J}_{RM}$ for both qutrit transitions which is assumed below unless otherwise stated.

For typical experimental parameters, the coupling strengths *J*’s are in the range of few to some tenths of MHz, while the energies Δ’s and *δ*’s are in the 10 GHz range. We choose realistic values of the experimental parameters so as to stay within the transmon regime^13^.

Apart from the intrinsic dynamics of the system, we will employ an external microwave (mw) field of (variable) frequency ${\omega }_{{\rm{mw}}}$ to drive the system. Physically, the driving can be applied through resonators 2 and 4 capacitively coupled to the outer qubits, and control lines 5 and 6 capacitively coupled to the qutrit, as shown in Fig. 1(a). The microwave field induces transitions between the qubit and qutrit states as described by the Hamiltonian

2 $$\begin{array}{rcl}{H}_{{\rm{mw}}} & = & \cos \,({\omega }_{{\rm{mw}}}t)\,({\Omega }_{L}{\sigma }_{L}^{+}+{\Omega }_{R}{\sigma }_{R}^{+}\\  &  & +\,{\Omega }_{1}|0\rangle \langle 1|+{\Omega }_{2}|1\rangle \langle 2|+{\rm{H}}.{\rm{c}}),\end{array}$$

where $\Omega$’s are the corresponding Rabi frequencies. Moreover, multifrequency pulses generated by an appropriate microwave source and directed to the qutrit via the control lines can be used to dynamically tune the qutrit transitions (see Supplementary Note 1 for the general treatment of transitions of a capacitively coupled qutrit). We note that unlike our qubits and qutrit, in flux qubits optical selection rules may depend on the magnetic flux^48^.

Our system can be used to achieve many quantum information tasks, examples of which are described below. The qutrit can encode a qubit in either states $(|0\rangle ,|1\rangle )$ or states $(|0\rangle ,|2\rangle )$. This is solely a matter of convenience and it is straightforward to toggle between these two encodings by applying a *π*-pulse on the $|1\rangle \leftrightarrow |2\rangle$ transition.

### Qutrit dissociation and entangled state preparation

We now discuss how to deterministically prepare entangled states in the setup, which is of great importance for quantum computation and information tasks^41,49^. In our system, we can employ the qutrit to deterministically prepare an entangled Bell state between the outer qubits $\frac{1}{\sqrt{2}}(|\downarrow \downarrow \rangle +|\uparrow \uparrow \rangle )$, as detailed below.

**Figure 3:**
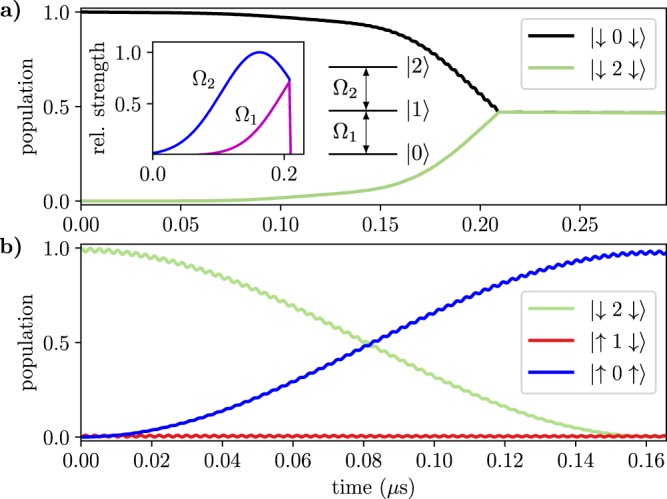
(**a**) Populations of states $|0\rangle$, $|1\rangle$ and $|2\rangle$ of the middle qutrit during the half STIRAP in the case of off-resonant qutrit levels and ${\rm{\max }}({\Omega }_{1,2})/2\pi =20\,{\rm{MHz}}$. The inset shows the envelopes of the mw pulses. (**b**) Dissociation of the initial state $|\downarrow 2\downarrow \rangle$ into the final state $|\uparrow 0\uparrow \rangle$ with the two-photon resonance ${\Delta }_{M}+{\delta }_{M}={\Delta }_{L}+{\Delta }_{R}$ while the intermediate states $|\uparrow 1\downarrow \rangle$, $|\downarrow 1\uparrow \rangle$ are off-resonant. See Supplementary Note 2 for the parameters used in the simulation. We also include finite coherence times as described in Methods.

First, we tune the energy levels of the qutrit to make its two transitions $|0\rangle \leftrightarrow |1\rangle$ and $|1\rangle \leftrightarrow |2\rangle$ non-resonant with the transitions $|\downarrow \rangle \leftrightarrow |\uparrow \rangle$ of the qubits, i.e., we require that ${\Delta }_{M}-{\Delta }_{\alpha }\gg {J}_{\alpha {M}_{01}}$ and ${\delta }_{M}-{\Delta }_{\alpha }\gg {J}_{\alpha {M}_{12}}$. Starting from the ground state $|0\rangle$, we produce the superposition state $\frac{1}{\sqrt{2}}(|0\rangle +|2\rangle )$ of the qutrit by external driving, employing the STIRAP (STImulated Raman Adiabatic Passage) sequence of pulses^50^, as has been also proposed^51^ and implemented^52^ in superconducting circuits before. Namely, we drive the transitions $|0\rangle \leftrightarrow |1\rangle$ and $|1\rangle \leftrightarrow |2\rangle$ with resonant mw-pulses of Rabi frequencies ${\Omega }_{1}$ and ${\Omega }_{2}$. The ${\Omega }_{2}$ pulse precedes the ${\Omega }_{1}$ pulse, and we adjust the overlap between the pulses so as to obtain the transfer to state $|2\rangle$ with minimal population of the intermediate state $|1\rangle$. The two pulses are suddenly turned off when their amplitudes are equal, resulting in the desired superposition state. The dynamics of the qutrit under the STIRAP driving is shown in Fig. 3a, with the inset showing the pulse sequence.

Next, the Bell state is obtained as $\frac{1}{\sqrt{2}}|\downarrow \downarrow \rangle \,(|0\rangle +|2\rangle )\to \frac{1}{\sqrt{2}}(|\downarrow \downarrow \rangle +|\uparrow \uparrow \rangle )\,|0\rangle$ via “dissociation” of the qutrit excitation $|2\rangle$ into two qubit excitations $|\uparrow \uparrow \rangle$. To this end, we set ${\Delta }_{M}+{\delta }_{M}={\Delta }_{L}+{\Delta }_{R}$ and choose ${\Delta }_{M}-{\Delta }_{\alpha } > {J}_{\alpha {M}_{01}}$ via tuning the frequencies of the outer qubits with flux control and the qutrit with the dynamical driving. Note that this condition applies when ${J}_{\alpha {M}_{01}}={J}_{\alpha {M}_{12}}$. If the exchange coefficients are different, as is normally the case, the qutrit is moved out of the two-photon resonance by unequal second order level shifts $|{J}_{\alpha {M}_{01}}{|}^{2}/({\Delta }_{M}-{\Delta }_{\alpha })\ne |{J}_{\alpha {M}_{12}}{|}^{2}/({\delta }_{M}-{\Delta }_{\alpha })$, which can be compensated for by adjusting Δ_*M*_ or *δ*_*M*_. Making the intermediate state $|1\rangle$ non-resonant precludes its population but prolongs the dissociation, which results in a more pronounced effect of the noise and relaxations. The dissociation dynamics $|\downarrow 2\downarrow \rangle \to |\uparrow 0\uparrow \rangle$ is shown in Fig. 3b.

Using the pairwise *ZZ*-interactions, the fully entangled three-particle *GHZ* (Green-Horne-Zeilinger) state $\frac{1}{\sqrt{2}}(|\downarrow 0\downarrow \rangle +|\uparrow 1\uparrow \rangle )$ can be obtained from the prepared Bell state $\frac{1}{\sqrt{2}}(|\downarrow 0\downarrow \rangle +|\uparrow 0\uparrow \rangle )$ by the external driving of the middle qutrit. To this end, we apply to the circuit a weak *π* pulse ${\Omega }_{1}$ which is resonant only for the $|\uparrow 0\uparrow \rangle \leftrightarrow |\uparrow 1\uparrow \rangle$ transition and non-resonant for the $|\downarrow 0\downarrow \rangle \leftrightarrow |\downarrow 1\downarrow \rangle$ transition, due to the *ZZ* interactions with the strengths ${J}_{\alpha M}^{(z)}\gg {\Omega }_{1}$.

Alternatively, we can encode a qubit in the $(|0\rangle ,|2\rangle )$ states of the qutrit and produce a different maximally entangled state $\frac{1}{\sqrt{2}}(|\downarrow 2\downarrow \rangle +|\uparrow 0\uparrow \rangle )$, equivalent to the *GHZ* state above. Starting from the simple initial state $|\downarrow 2\downarrow \rangle$, we use only the intrinsic system dynamics by tuning the parameters until ${\Delta }_{L}={\Delta }_{R}={\Delta }_{M}={\delta }_{M}$ and ${D}_{2}{J}_{\alpha M}^{(z)}=\frac{2\sqrt{6}{J}_{\alpha {M}_{01}}}{\sqrt{{(n\pi /{c}_{n})}^{2}-1}}$ for $n=1,2,3,\ldots$ and ${c}_{n}={\cos }^{-1}(\frac{{(-1)}^{n+1}}{8})$. Here, *n* is a parameter controlling at which oscillation between the states $|\downarrow 2\downarrow \rangle$ and $|\uparrow 0\uparrow \rangle$ their equal superposition is obtained (lower *n* is quicker). The disadvantage of this scheme is that it requires very precise tuning of the interactions ${J}_{\alpha M}^{(z)}$. In contrast, for the method above, only the frequencies have to be adjusted, which is easier using the dynamical tuning or an equivalent flux tuning. Further details are given in Supplementary Note 3.

### Toffoli and CCZ gates

The controlled-controlled not (ccnot) gate, also called the Toffoli gate, is a reversible and universal 3-bit gate for classical computation^35^. It performs a not (bit-flip) operation on the target bit if the two control bits are in state ‘1’, and does nothing otherwise. The Toffoli gate is an important element in many quantum algorithms, such as quantum error correction^53^ and Shor’s algorithm^54^. It has been implemented in systems ranging from trapped ions^55^ to superconducting circuits^40^, including a proposal for an implementation with static control optimized with machine learning^56^.

We can implement the ccnot gate with the left qubit and the middle qutrit acting as controls and the right qubit being the target. The state of the right qubit is then inverted only if the left qubit is in the spin up (excited) state $|\uparrow \rangle$ and the qutrit is in the ground state $|0\rangle$. The second control qubit is encoded in the qutrit states $|0\rangle$ and $|2\rangle$. The quantum ccnot gate is realized by first executing a double-controlled phase (ccz) gate that shifts the phase of the state $|\uparrow 0\uparrow \rangle$ by *π* (sign change) while nothing happens if either of the outer qubits are in the spin down state or if the qutrit is in state $|2\rangle$. The ccnot can then be obtained by the transformation: ccnot = ${\mathcal H}$ · ccz · ${\mathcal H}$, where ${\mathcal H}$ is the Hadamard gate that acts on the target qubit *R*. In practice, the Hadamard gate can be obtained by a *π*/2 rotation about the *y* axis.

**Figure 4:**
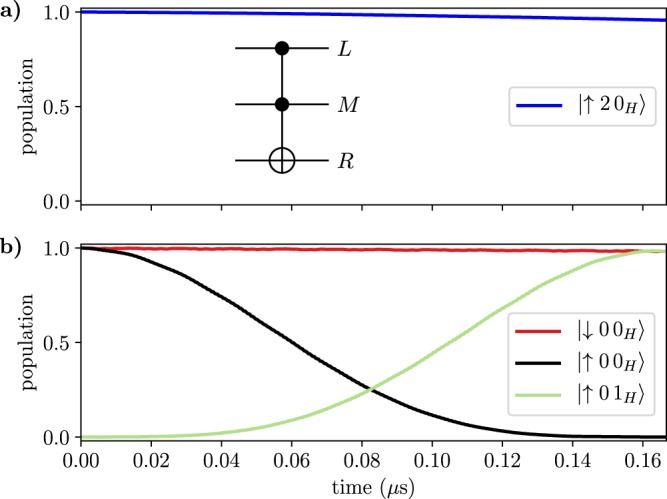
Numerical simulation of the implementation of the ccz gate in the rotating frame. The phase of the right qubit is flipped, $|{0}_{H}\rangle \to |{1}_{H}\rangle$, if the left qubit is in state $|\uparrow \rangle$ and the qutrit is in state $|0\rangle$, otherwise no change occurs as exemplified in (**a**) for the state $|\uparrow 2\,{0}_{H}\rangle$ and in (**b**) for the state $|\downarrow 0\,{0}_{H}\rangle$. A subsequent Hadamard gate on the right qubit will yield the desired ccnot gate. The standard circuit representation of the Toffoli gate is shown as an inset in the upper panel of the figure. See Supplementary Note 2 for the parameters used in the simulation.

The ccz gate is implemented by choosing suitable parameters such that the transitions between the qubit and qutrit states are non-resonant, while ${J}_{\alpha M}^{(z)}$ (>10 MHz) is large. We apply a weak microwave field on the qutrit transition $|\uparrow 0\uparrow \rangle \leftrightarrow |\uparrow 1\uparrow \rangle$ with the Rabi frequency ${\Omega }_{1}\ll {J}_{\alpha M}^{(z)}$. Because of the *ZZ* interactions, which yield a state-dependent frequency shift of the qutrit, the microwave field frequency can be chosen such that it is resonant only when both outer qubits are in the spin-up state. The microwave 2*π*-pulse then results in the transformation $|0\rangle \to i|1\rangle \to -\,|0\rangle$ that leads to the double conditional *π* phase change of (only) the state $|\uparrow 0\uparrow \rangle$. For simplicity, we have here used a standard square-pulse control. In a real-life implementation, a DRAG pulse^57^ or similar optimized pulses could be used, suppressing leakage to, and phase errors from, other levels and thus further improving the fidelity. In Fig. 4, we show the results of the numerical simulations of the ccz gate in the Hadamard basis for the right qubit (defined as $|{0}_{H}\rangle =|+\rangle =(|\downarrow \rangle +|\uparrow \rangle )/\sqrt{2}$ and $|{1}_{H}\rangle =|-\rangle =(|\downarrow \rangle -|\uparrow \rangle )/\sqrt{2}$). Subsequent application of the Hadamard gate to the right qubit will complete the ccnot gate. We note that because of the symmetry of the driving, we could have also chosen the qutrit state $|1\rangle$ instead of $|0\rangle$ as the ‘open’ state, but here we can view this merely as an ancillary state.

### Fredkin gate

**Figure 5:**
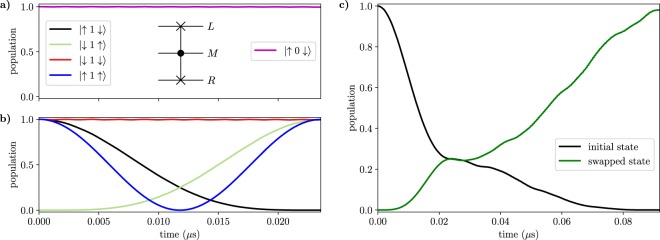
(**a** and **b**) Numerical simulations of the acswap (almost cswap) gate for different computational basis states, with the exchange interaction ${J}_{\alpha {M}_{12}}$ resonant for time $T=\pi /\sqrt{2}{J}_{\alpha {M}_{12}}$. The standard circuit representation of the Fredkin gate is shown as an inset in the top part of the figure. (**b**) Numerical simulation of the full cswap gate for the initial superposition state $[\cos ({\theta }_{1})|\uparrow \rangle +{{\rm{e}}}^{i{\varphi }_{1}}\,\sin ({\theta }_{1})|\downarrow \rangle ]$ $|1\rangle$ $[\cos ({\theta }_{2})|\uparrow \rangle +{{\rm{e}}}^{i{\varphi }_{2}}\,\sin ({\theta }_{2})|\downarrow \rangle ]$ with ${\theta }_{1}=\pi /4$, ${\varphi }_{1}=3\pi /4$, ${\theta }_{2}=3\pi /4$ and ${\varphi }_{2}={\varphi }_{1}$. In part 1, we perform the acswap operation during time ${T}_{1}=\pi /2{J}_{\alpha {M}_{01}}\simeq 0.025$ μs with the parameters as in panels (a and b). In part 2 we perform the ccz gate during time ${T}_{2}=2\pi /{\Omega }_{2}$ with the resonant mw field of frequency ${\omega }_{{\rm{mw}}}={\delta }_{M}-2{J}_{\alpha M}^{(z)}$. The full cswap fidelity of this state is >0.98, with finite coherence times included. See Supplementary Note 2 for the parameters used in the simulation.

Another classically universal 3-bit gate is the Fredkin gate, whose quantum analog is the controlled swap (cswap) gate. Its effect is to swap the states of the two qubits, $|\uparrow \downarrow \rangle \leftrightarrow |\downarrow \uparrow \rangle$, conditional upon the state of a control qubit, here encoded in the qutrit. We now use the two lowest states ($|0\rangle$ and $|1\rangle$) of the qutrit to encode the qubit such that the excited state $|1\rangle$ is ‘on’ and the ground state $|0\rangle$ is ‘off’. To realize cswap, we tune the energy levels of the qutrit such that the transition $|1\rangle \leftrightarrow |2\rangle$ is resonant with the qubit transitions $|\uparrow \rangle \leftrightarrow |\downarrow \rangle$, i.e. ${\Delta }_{L}\simeq {\delta }_{M}\simeq {\Delta }_{R}$. Simultaneously, the qutrit transition $|0\rangle \leftrightarrow |1\rangle$ is largely detuned, $|{\Delta }_{M}-{\Delta }_{L,R}|\gg {J}_{\alpha {M}_{01}}$. We then keep the resonance of the exchange interaction ${J}_{\alpha {M}_{12}}\gg {J}_{\alpha M}^{(z)}$ for time $T=\pi /\sqrt{2}{J}_{\alpha {M}_{12}}$. If the qutrit is in state $|0\rangle$, the qubits remain in their initial states due to absence of resonant transitions. But if the qutrit is in state $|1\rangle$, it would induce the swap between the qubit states, $|\uparrow 1\downarrow \rangle \leftrightarrow |\downarrow 1\uparrow \rangle$, via the resonant intermediate state $|\downarrow 2\downarrow \rangle$ involving the qutrit excitation. (Resonant swap between the qubits would also occur for the qutrit initially in state $|2\rangle$, with the intermediate state being $|\uparrow 1\uparrow \rangle$). This is illustrated in Fig. 5a.

As can also be seen in Fig. 5a, however, the initial state $|\downarrow 1\downarrow \rangle$ has trivial dynamics, unlike the rest of the swapped states which attain a *π* phase shift during the interaction time *T*. This means that we have a swap operation only up to a conditional phase for an arbitrary superposition input state. This is related to the phase shift of the swapped terms arising in the iswap gate, obtained by directly coupling two resonant qubits, which has recently attracted great interest^9,58^. In our case with the qutrit mediating the swap, only one state has a sign that needs correction, similarly to what Kivlichan *et al*. has recently called the “fermionic simulation gate”^59^. Because of this, we can easily mitigate this problem by using the ccz gate (see Subsection “Toffoli and CCZ gates”) to attain the *π* phase shift of state $|\downarrow 1\downarrow \rangle$ and obtain the correct cswap gate. In Fig. 5c we show the results of our numerical simulations of the complete cswap protocol, including the conditional resonant swap followed by the ccz gate with a total fidelity >98%. More detailed analysis is given in Supplementary Note 4.

We note that we could have equivalently performed the cswap gate between the two qubits via the resonant qutrit transition $|0\rangle \leftrightarrow |1\rangle$, while the other transition $|1\rangle \leftrightarrow |2\rangle$ is non-resonant. In our scheme, the qutrit has to play the role of control and thus our Fredkin gate is not a universal multi-qubit gate in itself. We could, however, imagine another qubit with controlled coupling to the qutrit as part of a larger universal circuit.

### Double-controlled holonomic gate

Another concept with importance to quantum computation^34^ is the implementation of general (non-abelian) one-qubit gates of the form (neglecting overall phase factors)

3 $$U=(\begin{array}{cc}{{\rm{e}}}^{i{\varphi }_{1}}\,\cos (\theta ) & {{\rm{e}}}^{i{\varphi }_{2}}\,\sin (\theta )\\ -{{\rm{e}}}^{-i{\varphi }_{2}}\,\sin (\theta ) & {{\rm{e}}}^{-i{\varphi }_{1}}\,\cos (\theta )\end{array}).$$

Together with a non-trivial (entangling) multi-qubit gate, they form a universal set of quantum gates^34^. We can implement the non-adiabatic one-qubit holonomic gate^44,60^ with our qutrit, choosing states $(|0\rangle ,|2\rangle )$ to encode the qubit. Such gates have the advantages of being robust to parameter fluctuations due to the geometric nature, without the limitations of long gate operation times required to satisfy the adiabatic requirement^61,62^. Holonomic gates have been implemented in a range of different systems^63,64^, and their stability has been well tested^65,66^. Choosing the same system parameters as for the ccz gate above, we use a driving scheme inspired by^63^ and thereby realize the single-qubit gate

4 $$U(\varphi ,\theta )=(\begin{array}{cc}\cos (\theta ) & {{\rm{e}}}^{i\varphi }\,\sin (\theta )\\ {{\rm{e}}}^{-i\varphi }\,\sin (\theta ) & -\cos (\theta )\end{array}),$$

with the computational qubit states as basis. This transformation is less general than (3), but it is still universal for one-qubit rotations.

We drive the two transitions $|0\rangle \leftrightarrow |1\rangle$ and $|1\rangle \leftrightarrow |2\rangle$ with the external fields having the same Gaussian envelope $\Omega (t)$ but different complex coupling amplitudes *a* and *b*, i.e. ${\Omega }_{1}(t)=a\Omega (t)$ and ${\Omega }_{2}(t)=b\Omega (t)$ in Equation (2), satisfying $|a{|}^{2}+|b{|}^{2}=1$. The pulse $\Omega (t)$ is turned on at time $t=0$ and turned off at $t=\tau$, such that we get a 2*π*-pulse, ${\int }_{0}^{\tau }\,\Omega (t)dt=2\pi$. Notice that this condition ensures that we end up with a closed path in parameter space and the gate is indeed holonomic. Starting with the qutrit in the ground state $|0\rangle$, we then obtain the final transformation $U(\varphi ,\theta )$ of Equation (4) acting on the qutrit states $|0\rangle ,|2\rangle$. Here *θ* and $\varphi$ are defined via ${{\rm{e}}}^{i\varphi }\,\tan (\theta /2)=a/b$.

By using the ${J}_{\alpha M}^{(z)}$ couplings to shift the qutrit frequencies, we can make the external driving field resonant or not, depending on the states of the outer qubits. This condition would then result in a controlled-controlled holonomic gate transforming the state of the qutrit according to (4) only when the outer (control) qubits are in e.g. the spin up state. We can show that this new gate is universal for quantum computing by first writing it in the three-qubit computational basis with $|6\rangle =|\uparrow 0\uparrow \rangle$ and $|7\rangle =|\uparrow 2\uparrow \rangle$, and the rest of the 8 basis states numbered from 0 to 5:5where ${\mathbb{I}}$ is the 6 × 6 identity matrix and the superscript c indicates that this is the controlled version of the holonomic gate. We now apply this transformation twice:6

This is equal to the famous Deutsch gate except for a factor *i* on the 2 × 2 rotation matrix. The Deutsch gate is universal for quantum computation^67^, and thus our double-controlled holonomic gate is also universal. Implementation of this gate have previously been proposed using Rydberg atoms^68^, albeit using three laser pulses instead of only two as in our case.

**Figure 6:**
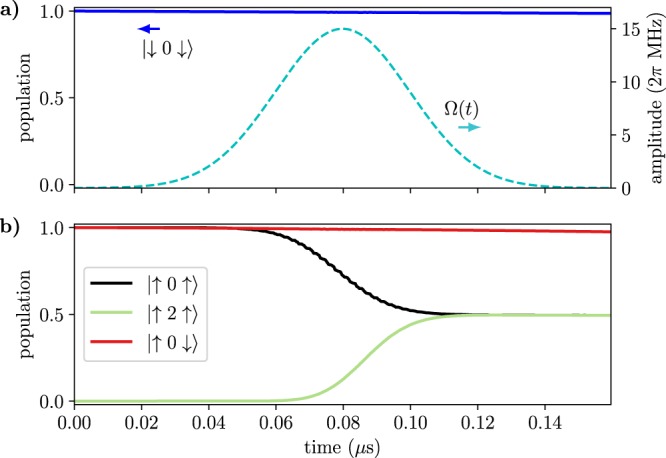
(**a** and **b**) Populations (left vertical axes) as function of time during the operation of the controlled-controlled holonomic gate in the case of $\theta =\pi /4$ and $\varphi =0$. Panel (a) also shows the envelope of the external fields plotted with dotted lines with the corresponding vertical axis on the right of the plot. See Supplementary Note 2 for the parameters used in the simulation.

In Fig. 6 we show the evolution of different initial states under gate operation. Evidently, the qutrit rotation is blocked when the left qubit is in the spin-down state or both qubits are in the spin down state, while the qutrit is rotated according to Equation (4) when both qubits are in the excited state.

**Figure 7:**
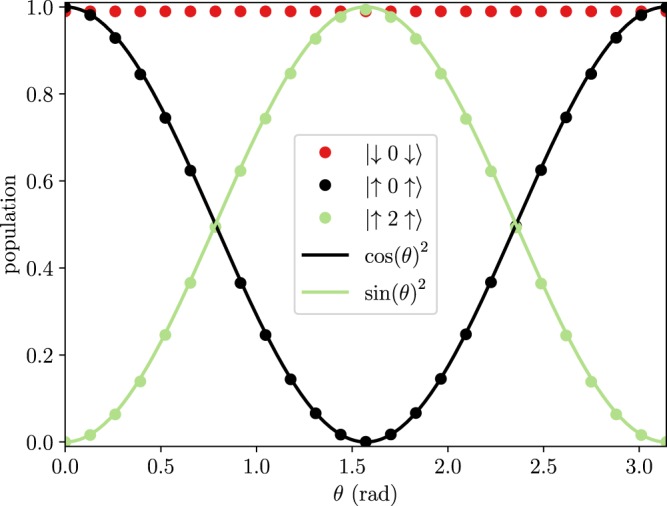
Populations of state versus *θ* ($\varphi =0$) after the application of the controlled-controlled holonomic gate to the initial state $|\uparrow 0\uparrow \rangle$ (black and green points) and $|\downarrow 0\uparrow \rangle$ (red points). See Supplementary Note 2 for the parameters used in the simulation.

In Fig. 7, we show the populations of the final states for various values of *θ*, while $\varphi =0$. The theoretical curves cos^2^ *θ* and sin^2^ *θ* from (4) are also shown and we observe a very good agreement. The final population of the blocked state $|\downarrow 0\uparrow \rangle$ is somewhat lower than expected primarily due to leakage to the other levels via a weak interaction with the external field, even though it is far from resonance. This leakage is also apparent in Fig. 6 and can potentially be reduced by employing pulse shaping techniques^57^.

## Discussion

To summarize, we have proposed a realistic superconducting circuit, consisting of a qutrit and two qubits, for efficient implementations of multi-qubit quantum gates. By utilizing the second excited state of the qutrit in the middle position, we proposed simple schemes for generating a maximally entangled Bell state of the outer qubits and a GHZ state of the qubits and the qutrit. Furthermore, our construction can implement several important quantum gates, such as the ccnot (Toffoli), and cswap (Fredkin) gates. We note that with qubits only, the theoretically most efficient realizations of the Fredkin and Toffoli gates each requires five two-qubit gates^38^. State of the art implementations of the Toffoli and CCZ gates using superconducting circuits have operation times ranging from 90 ns (with poor fidelity)^40^ to about 260 ns^69^. As for the Fredkin gate, we are not aware of an implementation with a superconducting circuit, but a hybrid scheme proposal has a gate execution time of 350 ns^70^. Using the current state of the art superconducting systems requiring 40 ns per two-qubit gate^71,72^, the total three-qubit gate time would be at least 200 ns. For comparison, our proposed scheme can complete the three-qubit operations in 100 ns. Thus, our results exemplify the flexibility and usefulness of qutrits for very efficient realizations of three-qubit gates, and demonstrate the potential of our circuit to serve as a basis for more complicated superconducting circuits.

Our scheme can implement in principle any controlled-controlled unitary operation on the qutrit. As an example, we have considered the double-controlled non-abelian holonomic quantum gate on a single qubit, which can be used to implement the three-qubit Deutsch gate in only two operations. This implementation is more effective than current proposals with Rydberg atoms^68^, while we are not aware of an implementation using superconducting circuits. We have implemented the holonomic gate as it is robust to parameter noise^61^ stemming from the geometric nature of this gate. The strategy of using such gates is known as holonomic quantum computation (HQC)^44^ and the universal non-abelian HQC (NHQC) generalization has since been performed by Sjöqvist *et al*.^46^. Here, three bare energy eigenstates are needed and are conveniently provided by the qutrit. A natural next step is to try to implement the two-qubit non-adiabatic holonomic quantum gate also suggested by Sjöqvist *et al*., requiring two nearest-neighbor qutrits. Such a gate could be possibly achieved by our circuit upon expanding the basis of one of the outer qubits. Together with the holonomic one-qubit gate, this would realize a universal set of holonomic gates.

Realizing qutrit-qutrit interactions would also open the possibility of implementing higher-order effective spin chains, such as the spin-one Haldane spin model^73,74^, especially if the coherence times of higher levels are further prolonged^75^.

Another possible use of qutrits and a circuit similar to the one proposed in this paper is the implementation of autonomous quantum error correction via engineered dissipation. With a relatively small increase in circuit complexity including three energy levels, an impressive increase in transmon coherence time was predicted in ref. ^76^.

## Methods

Consider the circuit with four connected superconducting islands with lumped element circuit shown in Fig. 1b. After obtaining the Lagrangian of the corresponding effective lumped element model system in the node flux picture, we perform a suitable change of coordinates, primarily mixing the two central flux node coordinates: ${\psi }_{1}={\varphi }_{a}+{\varphi }_{b}-2{\varphi }_{c}$, ${\psi }_{2}={\varphi }_{a}-{\varphi }_{b}$ and ${\psi }_{3}={\varphi }_{a}+{\varphi }_{b}-2{\varphi }_{d}$, where the $\varphi$s are the flux node variables shown in the circuit (in natural units). They represent the horizontal dipole mode between the left superconducting island and the two middle islands, the vertical dipole mode between the two middle islands, and the horizontal mode between the right island and the two middle islands, respectively. With this choice of coordinates, we obtain three effective nodes with the relevant degrees of freedom sequentially coupled via non-linear interactions. We truncate the outer nodes to the lowest two states, obtaining qubits, while for the middle node we instead choose to truncate its Hilbert space to the lowest three energy levels, obtaining a qutrit. All three degrees of freedom are in the transmon limit with the kinetic energy terms being much smaller than the potential energy terms. Finally, by transforming to a rotating frame and making a rotating wave approximation to eliminate the fast oscillating terms, we obtain an effective Hamiltonian for the system of two qubits each coupled to the qutrit (see Supplementary Note 1 for the full derivation). This Hamiltonian is given in (1).

The drive line terms are added to the non-truncated Lagrangian as externally varied flux nodes and a transformation to an appropriate frame rotating with the external field is performed. This transformation mixes the variables and after additional rotating wave approximations, the desired external part of the Hamiltonian is obtained along with modifications on the energy parameters, i.e. the AC-Stark shifts, which can be used for tuning the qubits and qutrit in and out of resonance.

We simulate the dissipative dynamics of the system numerically, with the relaxation and decoherence times set to *T*_1_ = 31 μs and *T*_2_ = 35 μs respectively, based on recent studies^15,75,77^ (we use the Python QuTip package^78^, and relaxations are implemented by the simple built-in collapse operator functionality). The parameters of the Hamiltonian used in the numerical simulations are all obtained from realistic experimental circuit parameters, as detailed in Supplementary Note 1, and are listed in Supplementary Note 2 for each implementation.

## Supplementary information

Supplementary information

## Data Availability

The data that support the findings of this study are available from N.T.Z. upon request.
