# Supplementary material for: Realization of efficient quantum gates with a superconducting qubit-qutrit circuit
Source: Sci Rep. 2019 Sep 16;9:13389. doi: 10.1038/s41598-019-49657-1 (PMC6746868; doi:10.1038/s41598-019-49657-1)
Supplement: Supplementary file 1 — Supplementary information [file 41598_2019_49657_MOESM1_ESM.pdf]

Realization of efficient quantum gates with a superconducting qubit-qutrit circuit - Supplementary Information

T. Bækkegaard, L.B. Kristensen, N.J.S. Loft, C.K. Andersen, D. Petrosyan, and N.T. Zinner

April 30, 2019

Supplementary Note 1: Derivation of the Hamiltonian for the circuit

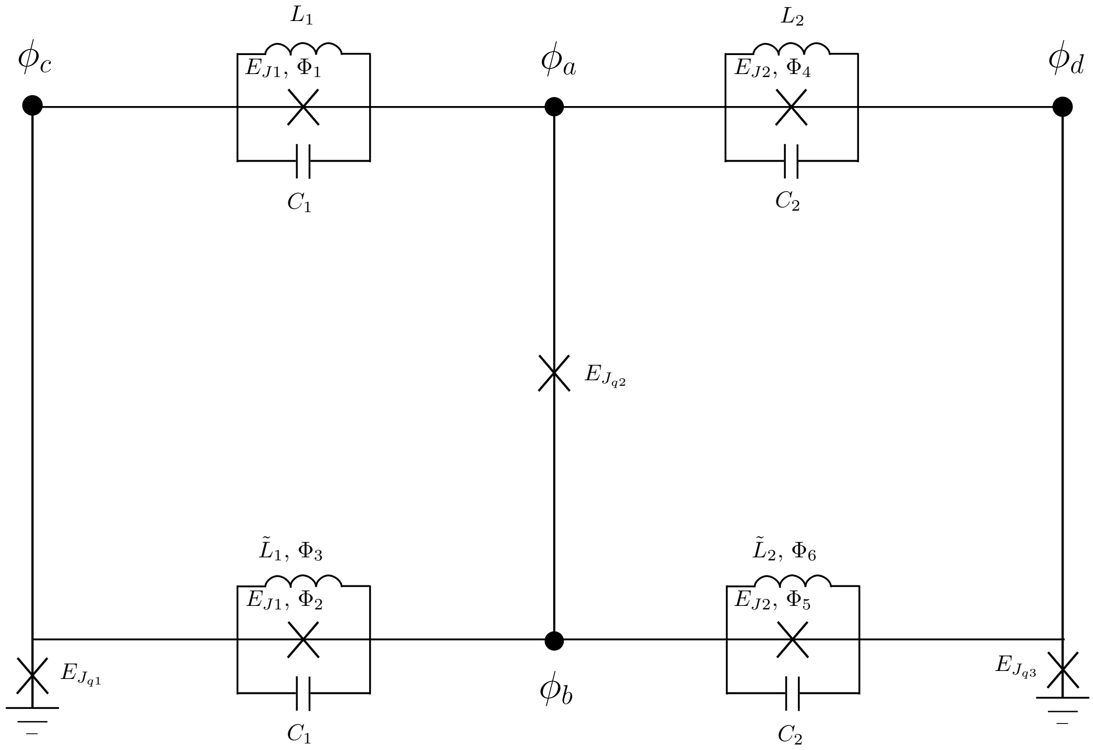

Supplementary Figure 1: The superconducting circuit and the corresponding parameters describing the properties of the components. Indicated are also the nodes and the corresponding fluxes.

We are considering the effective circuit in fig. 1 and want to show that the low-energy degrees of freedom of this circuit constitutes three qubits(qutrits) with a Heisenberg XXZ-interaction. We start by writing down the Lagrangian of the system in the node flux picturewith the closure branches being the two lower horizontal branches (each splitting into

three branches with different circuit elements). The resulting Lagrangian is:

$$\begin{aligned} L = & \frac{C_1}{2}\left(\dot{\phi}_a - \dot{\phi}_c\right)^2 + \frac{C_1}{2}\left(\dot{\phi}_b - \dot{\phi}_c\right)^2 - \frac{1}{2L_1}\left(\phi_a - \phi_c\right)^2 - \frac{1}{2\tilde{L}_1}\left(\phi_b - \phi_c + \Phi_3\right)^2 \\ & + \frac{C_2}{2}\left(\dot{\phi}_a - \dot{\phi}_d\right)^2 + \frac{C_2}{2}\left(\dot{\phi}_b - \dot{\phi}_d\right)^2 - \frac{1}{2L_2}\left(\phi_a - \phi_d\right)^2 - \frac{1}{2\tilde{L}_2}\left(\phi_b - \phi_d + \Phi_6\right)^2 \\ & + E_{J1}\left[\cos(\phi_a - \phi_c + \Phi_1) + \cos(\phi_b - \phi_c + \Phi_2)\right] \\ & + E_{J2}\left[\cos(\phi_a - \phi_d + \Phi_4) + \cos(\phi_b - \phi_d + \Phi_5)\right] \\ & + E_{J_{q1}}\cos(\phi_c) + E_{J_{q2}}\cos(\phi_a - \phi_b) + E_{J_{q3}}\cos(\phi_d). \end{aligned} \tag{1}$$

Defining $\Phi_{\Sigma 1} = \Phi_1 + \Phi_2$ and assuming $\Phi_1 - \Phi_2 = 0$, we can rewrite the third line using trigonometric identities:

$$2E_{J1}\cos\left(\frac{\phi_a + \phi_b - 2\phi_c + \Phi_{\Sigma 1}}{2}\right)\cos\left(\frac{\phi_a - \phi_b}{2}\right). \tag{2}$$

We will stay in the transmon regime , where the potential terms are much larger than the kinetic terms. This means that we can assume to be close to the potential minimum, approximated to first order by a harmonic oscillator. Thus, we will later rewrite the Hamiltonian in terms of the bosonic step operators related to the harmonic part of the Hamiltonian. We thereafter employ a rotating wave approximation, removing all terms with odd dependence of the node flux variables since these will be energy non-conserving. Specifically these will, after the truncation to the lowest energy levels, represent spontaneous excitation terms. From the Taylor expansion of trigonometric functions, we notice that we can further simplify the expression (2) to the following form using more trigonometric identities:

$$2E_{J1}\cos\left(\frac{\Phi_{\Sigma 1}}{2}\right)\cos\left(\frac{\phi_a + \phi_b - 2\phi_c}{2}\right)\cos\left(\frac{\phi_a - \phi_b}{2}\right). \tag{3}$$

Making the same kind of definition for the fourth term, i.e. $\Phi_{\Sigma 2} = \Phi_4 + \Phi_5$ and assuming $\Phi_4 - \Phi_5 = 0$, we can make the same simplification here. We can also ignore the terms dependent on Φ_3 and Φ_6 as these will again only be irrelevant offset terms or have an odd dependence on the node fluxes. The Lagrangian becomes

$$\begin{aligned} L = & \frac{C_1}{2}\left(\dot{\phi}_a - \dot{\phi}_c\right)^2 + \frac{C_1}{2}\left(\dot{\phi}_b - \dot{\phi}_c\right)^2 - \frac{1}{2L_1}\left(\phi_a - \phi_c\right)^2 - \frac{1}{2\tilde{L}_1}\left(\phi_b - \phi_c\right)^2 \\ & + \frac{C_2}{2}\left(\dot{\phi}_a - \dot{\phi}_d\right)^2 + \frac{C_2}{2}\left(\dot{\phi}_b - \dot{\phi}_d\right)^2 - \frac{1}{2L_2}\left(\phi_a - \phi_d\right)^2 - \frac{1}{2\tilde{L}_2}\left(\phi_b - \phi_d\right)^2 \\ & + 2E_{J1}\cos\left(\frac{\Phi_{\Sigma 1}}{2}\right)\cos\left(\frac{\phi_a + \phi_b - 2\phi_c}{2}\right)\cos\left(\frac{\phi_a - \phi_b}{2}\right) \\ & + 2E_{J2}\cos\left(\frac{\Phi_{\Sigma 2}}{2}\right)\cos\left(\frac{\phi_a + \phi_b - 2\phi_d}{2}\right)\cos\left(\frac{\phi_a - \phi_b}{2}\right) \\ & + E_{J_{q1}}\cos(\phi_c) + E_{J_{q2}}\cos(\phi_a - \phi_b) + E_{J_{q3}}\cos(\phi_d). \end{aligned}$$

The next step is defining a suitable set of variables. Inspired by the way the ϕ_i's enter the cosines above, we choose the following:

$$\begin{aligned} \psi_1 &= \phi_a + \phi_b - 2\phi_c \\ \psi_2 &= \phi_a - \phi_b \\ \psi_3 &= \phi_a + \phi_b - 2\phi_d \\ \psi_{\mathrm{CM}} &= \phi_a + \phi_b\ . \end{aligned} \tag{4}$$

In terms of the new variables after expansion of the brackets and collection of terms, the Lagrangian is:

$$\begin{aligned}
L = &\frac{C_1}{4}\dot{\psi_1}^2 - \left(\frac{1}{8L_1} + \frac{1}{8\tilde{L}_1}\right)\psi_1{}^2 + E_{J_{q1}}\cos\left(\frac{\psi_1 - \psi_{CM}}{2}\right) \\
&+ \left(\frac{C_1}{4} + \frac{C_2}{4}\right)\dot{\psi_2}^2 - \left(\frac{1}{8L_1} + \frac{1}{8\tilde{L}_1} + \frac{1}{8L_2} + \frac{1}{8\tilde{L}_2}\right)\psi_2{}^2 + E_{J_{q2}}\cos(\psi_2) \\
&+ \frac{C_2}{4}\dot{\psi_3}^2 - \left(\frac{1}{8L_2} + \frac{1}{8\tilde{L}_2}\right)\psi_3{}^2 + E_{J_{q3}}\cos\left(\frac{\psi_3 - \psi_{CM}}{2}\right) \\
&- \left(\frac{1}{8L_1} - \frac{1}{8\tilde{L}_1}\right)\psi_1\psi_2 + 2E_{J1}\cos\left(\frac{\Phi_{\Sigma 1}}{2}\right)\cos\left(\frac{\psi_1}{2}\right)\cos\left(\frac{\psi_2}{2}\right) \\
&- \left(\frac{1}{8L_2} - \frac{1}{8\tilde{L}_2}\right)\psi_2\psi_3 + 2E_{J2}\cos\left(\frac{\Phi_{\Sigma 2}}{2}\right)\cos\left(\frac{\psi_2}{2}\right)\cos\left(\frac{\psi_3}{2}\right) .
\end{aligned}$$

The conjugate momenta can be found from the usual definition $p_i = \frac{\partial L}{\partial \dot{\psi}_i}$:

$$\begin{aligned}
p_1 &= \frac{C_1}{2}\dot{\psi}_1 \\
p_2 &= \left(\frac{C_1}{2} + \frac{C_2}{2}\right)\dot{\psi}_2 \\
p_3 &= \frac{C_2}{2}\dot{\psi}_3 \\
p_{CM} &= 0 .
\end{aligned} \tag{5}$$

Notice that the conjugate momentum of ψ_{CM} is zero, and thus the variable can be seen as purely a constraint variable without a kinetic term. We will thus ignore ψ_{CM} from now on.

Now performing a Legendre transforming to the Hamiltonian via the relation $H = \sum_i \dot{\psi}_i p_i - L$, we obtain:

$$\begin{aligned}
H = &\frac{1}{C_1}p_1{}^2 + \left(\frac{1}{8L_1} + \frac{1}{8\tilde{L}_1}\right)\psi_1{}^2 - E_{J_{q1}}\cos\left(\frac{\psi_1}{2}\right) \\
&+ \frac{1}{C_1 + C_2}p_2{}^2 + \left(\frac{1}{8L_1} + \frac{1}{8\tilde{L}_1} + \frac{1}{8L_2} + \frac{1}{8\tilde{L}_2}\right)\psi_2{}^2 - E_{J_{q2}}\cos(\psi_2) \\
&+ \frac{1}{C_2}p_3{}^2 + \left(\frac{1}{8L_2} + \frac{1}{8\tilde{L}_2}\right)\psi_3{}^2 - E_{J_{q3}}\cos\left(\frac{\psi_3}{2}\right) \\
&+ \left(\frac{1}{8L_1} - \frac{1}{8\tilde{L}_1}\right)\psi_1\psi_2 - 2E_{J1}\cos\left(\frac{\Phi_{\Sigma 1}}{2}\right)\cos\left(\frac{\psi_1}{2}\right)\cos\left(\frac{\psi_2}{2}\right) \\
&+ \left(\frac{1}{8L_2} - \frac{1}{8\tilde{L}_2}\right)\psi_2\psi_3 - 2E_{J2}\cos\left(\frac{\Phi_{\Sigma 2}}{2}\right)\cos\left(\frac{\psi_2}{2}\right)\cos\left(\frac{\psi_3}{2}\right) .
\end{aligned}$$

The cosines are now expanded to fourth order in the flux variables:

$$\begin{aligned}
\cos(x) &\simeq 1 - \frac{1}{2}x^2 + \frac{1}{24}x^4 \\
\cos\left(\frac{x}{2}\right) &\simeq 1 - \frac{1}{8}x^2 + \frac{1}{384}x^4 \\
\cos\left(\frac{x_1}{2}\right)\cos\left(\frac{x_2}{2}\right) &\simeq 1 - \frac{1}{8}x_1{}^2 + \frac{1}{384}x_1{}^4 - \frac{1}{8}x_2{}^2 + \frac{1}{384}x_2{}^4 + \frac{1}{64}x_1{}^2x_2{}^2 .
\end{aligned} \tag{6}$$

This approximation neglects six-order terms and higher. Using first-order perturbation theory on the sixth-order term from $\cos(\psi_2)$ above, we get a correction to the final ground state energy of the corresponding qubit of $E^{(1)}/(2\pi) \simeq 31\,\text{kHz}$. This can safely be ignored compared to the usual transmon energies in the order of $10\,\text{GHz}$. Also, since we make sure to stay in the transmon limit with the potential terms much higher than the kinetic terms, we will always stay near very small values of the transmon phase drops, which is the physical interpretation of the flux variables, ψ_i. This makes, up to irrelevant constant terms,

$$
\begin{aligned}
H = &\frac{1}{C_1}{p_1}^2 + \alpha_1{\psi_1}^2 - \beta_1{\psi_1}^4 \\
&+ \frac{1}{C_1+C_2}{p_2}^2 + \alpha_2{\psi_2}^2 - \beta_2{\psi_2}^4 \\
&+ \frac{1}{C_2}{p_3}^2 + \alpha_3{\psi_3}^2 - \beta_3{\psi_3}^4 \\
&+ \left(\frac{1}{8L_1} - \frac{1}{8\tilde{L}_1}\right)\psi_1\psi_2 - \frac{E_{J1}}{32}\cos\left(\frac{\Phi_{\Sigma 1}}{2}\right){\psi_1}^2{\psi_2}^2 \\
&+ \left(\frac{1}{8L_2} - \frac{1}{8\tilde{L}_2}\right)\psi_2\psi_3 - \frac{E_{J2}}{32}\cos\left(\frac{\Phi_{\Sigma 2}}{2}\right){\psi_2}^2{\psi_3}^2,
\end{aligned}
$$

where

$$
\begin{aligned}
\alpha_1 &= \frac{1}{8L_1} + \frac{1}{8\tilde{L}_1} + \frac{E_{J_{q1}}}{8} + \frac{E_{J1}}{4}\cos\left(\frac{\Phi_{\Sigma 1}}{2}\right) \\
\beta_1 &= \frac{E_{J_{q1}}}{384} + \frac{E_{J1}}{192}\cos\left(\frac{\Phi_{\Sigma 1}}{2}\right) \\
\alpha_2 &= \frac{1}{8L_1} + \frac{1}{8\tilde{L}_1} + \frac{1}{8L_2} + \frac{1}{8\tilde{L}_2} + \frac{E_{J_{q2}}}{2} + \frac{E_{J1}}{4}\cos\left(\frac{\Phi_{\Sigma 1}}{2}\right) + \frac{E_{J2}}{4}\cos\left(\frac{\Phi_{\Sigma 2}}{2}\right) \\
\beta_2 &= \frac{E_{J_{q2}}}{24} + \frac{E_{J1}}{192}\cos\left(\frac{\Phi_{\Sigma 1}}{2}\right) + \frac{E_{J2}}{192}\cos\left(\frac{\Phi_{\Sigma 2}}{2}\right) \\
\alpha_3 &= \frac{1}{8L_2} + \frac{1}{8\tilde{L}_2} + \frac{E_{J_{q3}}}{8} + \frac{E_{J2}}{4}\cos\left(\frac{\Phi_{\Sigma 2}}{2}\right) \\
\beta_3 &= \frac{E_{J_{q3}}}{384} + \frac{E_{J2}}{192}\cos\left(\frac{\Phi_{\Sigma 2}}{2}\right).
\end{aligned}
\tag{7}
$$

Taking the anharmonicity (fourth-order terms) as a perturbation, we now choose the bosonic raising and lowering operators related to the harmonic parts of the Hamiltonian (the p^2 and ψ^2 -terms) above as usual (This perturbation is essential for the later truncation, since the resulting anharmonicity of the energy spacing between the levels allows us to only consider the lowest states):

$$
\begin{aligned}
\psi_1 &= \frac{1}{(4\alpha_1 C_1)^{\frac{1}{4}}}\left(b_1^\dagger + b_1\right) \\
\psi_2 &= \frac{1}{(4\alpha_2 (C_1+C_2))^{\frac{1}{4}}}\left(b_2^\dagger + b_2\right) \\
\psi_3 &= \frac{1}{(4\alpha_3 C_2)^{\frac{1}{4}}}\left(b_3^\dagger + b_3\right).
\end{aligned}
\tag{8}
$$

We end up with the rewritten Hamiltonian

$$
\begin{aligned}
H = &\sqrt{\frac{4\alpha_1}{C_1}}\left(b_1^\dagger b_1 + \frac{1}{2}\right) - \frac{\beta_1}{4\alpha_1 C_1}\left(b_1^\dagger + b_1\right)^4 \\
&+ \sqrt{\frac{4\alpha_2}{C_1+C_2}}\left(b_2^\dagger b_2 + \frac{1}{2}\right) - \frac{\beta_2}{4\alpha_2(C_1+C_2)}\left(b_2^\dagger + b_2\right)^4 \\
&+ \sqrt{\frac{4\alpha_3}{C_2}}\left(b_3^\dagger b_3 + \frac{1}{2}\right) - \frac{\beta_3}{4\alpha_3 C_2}\left(b_3^\dagger + b_3\right)^4 \\
&+ \left(\frac{1}{8L_1} - \frac{1}{8\tilde{L}_1}\right)\frac{1}{2(\alpha_1\alpha_2 C_1(C_1+C_2))^{\frac{1}{4}}}\left(b_1^\dagger + b_1\right)\left(b_2^\dagger + b_2\right) \\
&- \frac{E_{J1}}{128}\cos\left(\frac{\Phi_{\Sigma 1}}{2}\right)\frac{1}{\sqrt{\alpha_1\alpha_2 C_1(C_1+C_2)}}\left(b_1^\dagger + b_1\right)^2\left(b_2^\dagger + b_2\right)^2 \\
&+ \left(\frac{1}{8L_2} - \frac{1}{8\tilde{L}_2}\right)\frac{1}{2(\alpha_2\alpha_3 C_2(C_1+C_2))^{\frac{1}{4}}}\left(b_2^\dagger + b_2\right)\left(b_3^\dagger + b_3\right) \\
&- \frac{E_{J2}}{128}\cos\left(\frac{\Phi_{\Sigma 2}}{2}\right)\frac{1}{\sqrt{\alpha_2\alpha_3 C_2(C_1+C_2)}}\left(b_2^\dagger + b_2\right)^2\left(b_3^\dagger + b_3\right)^2.
\end{aligned}
\tag{9}
$$

We could now in principle map this to a spin model by using the anharmonicity to truncate to the lowest two/three energy eigenstates (e.g. $b_i^\dagger + b_i \mapsto \sigma_i^x$ for a qubit) and after using the rotating wave approximation end up with a Hamiltonian with the desired form. This would have the drawback of being static, i.e. once the circuit is built to certain specifications the experimental parameters are fixed and the energy levels and coupling strengths can not be adjusted significantly afterwards. We will introduce a dynamical tuning by adding an external driving field, effectively mixing the first and second excited state of the middle (ψ_2) degree of freedom and thereby changing the energy levels and coupling strengths.

Adding an effective external energy level tuning

We now imagine control lines connecting to the nodes a and b as in the main text. These can then be used to drive the middle degree of freedom ψ_2 using external fields. Specifically, we let the control line 5 connect to the node with flux ϕ_a in Supplemetary Fig. 1 through the capacitance C_{ext} and drive an external field ϕ_{ext}. Similarly, we apply an external field $\phi_{-\text{ext}}$ through control line 6 connected to the node flux ϕ_b through the same capacitance C_{ext}. The following extra term will appear in the Lagrangian:

$$L_{\text{ext}} = \frac{C_{\text{ext}}}{2}\left(\dot{\phi}_a - \dot{\phi}_{\text{ext}}\right)^2 + \frac{C_{\text{ext}}}{2}\left(\dot{\phi}_b - \dot{\phi}_{-\text{ext}}\right)^2. \tag{10}$$

This can be rewritten to

$$L_{\text{ext}} = \frac{C_{\text{ext}}}{4}\left[\left(\dot{\phi}_a + \dot{\phi}_b - \dot{\phi}_{-\text{ext}} - \dot{\phi}_{\text{ext}}\right)^2 + \left(\dot{\phi}_a - \dot{\phi}_b + \dot{\phi}_{-\text{ext}} - \dot{\phi}_{\text{ext}}\right)^2\right].$$

Assuming $\phi_{\text{ext}} = -\phi_{-\text{ext}} = A_{\text{ext}}\sin(\omega_{\text{ext}}t)$ and transforming to the ψ-coordinates, this reduces to

$$\begin{aligned} L_{\text{ext}} &= \frac{C_{\text{ext}}}{4}\left[\dot{\psi}_{CM}^2 + \left(\dot{\psi}_2 - 2A_{\text{ext}}\omega_{\text{ext}}\cos(\omega_{\text{ext}}t)\right)^2\right] \\ &= \frac{C_{\text{ext}}}{4}\dot{\psi}_{CM}^2 + \frac{C_{\text{ext}}}{4}\dot{\psi}_2^2 + C_{\text{ext}}A_{\text{ext}}^2\omega_{\text{ext}}^2\cos(\omega_{\text{ext}}t)^2 - C_{\text{ext}}\dot{\psi}_2 A_{\text{ext}}\omega_{\text{ext}}\cos(\omega_{\text{ext}}t). \end{aligned} \tag{11}$$

The first term here is an apparently problematic kinetic term for what has so far been a constraint variable. This can, though, be constructed to have a spacing between the energy levels that is very far from the spacing of the other degrees of freedom and ψ_{CM} can thus be ignored in spite of this term. The second term is another kinetic term for the ψ_2-variable, the third term is an offset term, and the fourth and last term is an interaction term between ψ_2 and the external field. It is this last term that will be useful for driving transitions between, and hence coupling of, the first and second excited levels of ψ_2. This will allow us to tune the position of the two levels through non-crossing depending on how much we mix the states.

Including the above addition, the Lagrangian related purely to the ψ_2-degree of freedom is the following:

$$L_2 = \frac{C_1 + C_2 + C_{\text{ext}}}{4}\dot{\psi}_2{}^2 - \left(\frac{1}{8L_1} + \frac{1}{8\tilde{L}_1} + \frac{1}{8L_2} + \frac{1}{8\tilde{L}_2}\right)\psi_2{}^2 + E_{J_{q2}}\cos(\psi_2) - C_{\text{ext}}A_{\text{ext}}\omega_{\text{ext}}\cos(\omega_{\text{ext}}t)\dot{\psi}_2,$$

with all other terms being either purely related to ψ_1 or ψ_3 or interaction terms. The corresponding conjugated momentum is also altered slightly:

$$p_2 = \frac{C_1 + C_2 + C_{\text{ext}}}{2}\dot{\psi}_2 - C_{\text{ext}}A_{\text{ext}}\omega_{\text{ext}}\cos(\omega_{\text{ext}}t), \tag{12}$$

thus

$$\dot{\psi}_2 = \frac{2}{C_1 + C_2 + C_{\text{ext}}}\left(p_2 + C_{\text{ext}}A_{\text{ext}}\omega_{\text{ext}}\cos(\omega_{\text{ext}}t)\right). \tag{13}$$

The related Hamiltonian is therefore, ignoring any offset terms:

$$\begin{aligned} H_2 = &\frac{p_2{}^2}{C_1 + C_2 + C_{\text{ext}}} + \frac{2C_{\text{ext}}A_{\text{ext}}\omega_{\text{ext}}\cos(\omega_{\text{ext}}t)}{C_1 + C_2 + C_{\text{ext}}}p_2 \\ &+ \left(\frac{1}{8L_1} + \frac{1}{8\tilde{L}_1} + \frac{1}{8L_2} + \frac{1}{8\tilde{L}_2}\right)\psi_2{}^2 - E_{J_{q2}}\cos(\psi_2). \end{aligned}$$

Performing the same expansions as before addition of the extra capacitances and external fields gives a few extra ψ_2-terms from the interaction terms. Focusing on only the resulting terms containing only ψ_2 or p_2, we get:

$$H_2 = \frac{1}{C_1 + C_2 + C_{\text{ext}}}p_2{}^2 + \alpha_2\psi_2{}^2 - \beta_2\psi_2{}^4 + \frac{2C_{\text{ext}}A_{\text{ext}}\omega_{\text{ext}}\cos(\omega_{\text{ext}}t)}{C_1 + C_2 + C_{\text{ext}}}p_2, \tag{14}$$

where α_2 and β_2 are defined in (7). We once again introduce the bosonic step-operators related to the harmonic part of H_2:

$$\begin{aligned}\psi_2 &= \frac{1}{(4\alpha_2(C_1+C_2+C_{\text{ext}}))^{\frac{1}{4}}}\left(b_2^\dagger + b_2\right)\\ p_2 &= i\frac{(4\alpha_2(C_1+C_2+C_{\text{ext}}))^{\frac{1}{4}}}{2}\left(b_2^\dagger - b_2\right),\end{aligned} \tag{15}$$

which makes

$$H_2 = T_1 b_2^\dagger b_2 - T_2\left(b_2^\dagger + b_2\right)^4 + iT_{\text{ext}}\cos\left(\omega_{\text{ext}}t\right)\left(b_2^\dagger - b_2\right), \tag{16}$$

where

$$\begin{aligned}T_1 &= \sqrt{\frac{4\alpha_2}{C_1+C_2+C_{\text{ext}}}}\\ T_2 &= \frac{\beta_2}{4\alpha_2(C_1+C_2+C_{\text{ext}})}\\ T_{\text{ext}} &= C_{\text{ext}}A_{\text{ext}}\omega_{\text{ext}}\frac{(4\alpha_2(C_1+C_2+C_{\text{ext}}))^{\frac{1}{4}}}{C_1+C_2+C_{\text{ext}}}.\end{aligned} \tag{17}$$

We wish to diagonalize this Hamiltonian to investigate the effect of T_{ext} on the spectrum.

Truncating the middle degree of freedom

We will now investigate the dynamical tuning of the spectrum by first truncating the "internal" Hamiltonian for the ψ_2 degree of freedom, corresponding to setting $A_{\text{ext}} = 0$, to the three lowest degrees of freedom, i.e. find the qutrit eigenstates. We will then add the external field and transform to a frame rotating with the external field, wherein we can see the effective mixing of the qutrit eigenstates. Lastly, we shift basis and use these driven states in the rotating frame as our qutrit eigenstates.

We start by diagonalizing the internal Hamiltonian of the middle degree of freedom "$H_{2,0}$", i.e. the first two terms in (16): In the basis of the three lowest simple harmonic oscillator states, which is chosen since we wish to end up with a qutrit in the end, we represent (up to irrelevant offset terms proportional to the identity)

$$b_2^\dagger \sim \begin{pmatrix}0 & 0 & 0\\ 1 & 0 & 0\\ 0 & \sqrt{2} & 0\end{pmatrix}, \qquad b_2 \sim \begin{pmatrix}0 & 1 & 0\\ 0 & 0 & \sqrt{2}\\ 0 & 0 & 0\end{pmatrix}. \tag{18}$$

This truncation is also done for all terms in (16), after using the canonical commutation relation $[b, b^\dagger] = 1$ to transform to normal ordering form:

$$b_2^\dagger b_2 \sim \begin{pmatrix}0 & 0 & 0\\ 0 & 1 & 0\\ 0 & 0 & 2\end{pmatrix}, \qquad \left(b_2^\dagger + b_2\right)^4 \sim \begin{pmatrix}0 & 0 & 6\sqrt{2}\\ 0 & 12 & 0\\ 6\sqrt{2} & 0 & 36\end{pmatrix},$$

and

$$b_2^\dagger - b_2 \sim \begin{pmatrix}0 & -1 & 0\\ 1 & 0 & -\sqrt{2}\\ 0 & \sqrt{2} & 0\end{pmatrix}$$

Inserting this in the first two terms in equation (16), we get the Hamiltonian

$$H_{2,0} \sim \begin{pmatrix}0 & 0 & -6\sqrt{2}T_2\\ 0 & T_1 - 12T_2 & 0\\ -6\sqrt{2}T_2 & 0 & 2T_1 - 36T_2\end{pmatrix},$$

which has the eigenenergies:

$$\begin{aligned}E_0 &= T_1 - 18T_2 - \sqrt{(T_1 - 18T_2)^2 + 72{T_2}^2}\\ E_1 &= T_1 - 12T_2\\ E_2 &= T_1 - 18T_2 + \sqrt{(T_1 - 18T_2)^2 + 72{T_2}^2}\end{aligned} \tag{19}$$

with the corresponding eigenstates:

$$
\begin{aligned}
\left|\tilde{0}\right\rangle &= \frac{1}{\sqrt{72{T_2}^2 + {E_0}^2}} \begin{pmatrix} 6\sqrt{2}T_2 \\ 0 \\ -E_0 \end{pmatrix} \\
\left|\tilde{1}\right\rangle &= \begin{pmatrix} 0 \\ 1 \\ 0 \end{pmatrix} \\
\left|\tilde{2}\right\rangle &= \frac{1}{\sqrt{72{T_2}^2 + {E_2}^2}} \begin{pmatrix} -6\sqrt{2}T_2 \\ 0 \\ E_2 \end{pmatrix}.
\end{aligned}
\tag{20}
$$

Now turning on the full external field, the full Hamiltonian H_2 can be expressed as

$$
H_2 = E_0 \left|\tilde{0}\middle\rangle\!\middle\langle\tilde{0}\right| + E_1 \left|\tilde{1}\middle\rangle\!\middle\langle\tilde{1}\right| + E_2 \left|\tilde{2}\middle\rangle\!\middle\langle\tilde{2}\right| + \frac{T_{\text{ext}}}{2} \left(\mathrm{e}^{i\omega_{\text{ext}}t} + \mathrm{e}^{-i\omega_{\text{ext}}t}\right) \begin{pmatrix} 0 & -i & 0 \\ i & 0 & -\sqrt{2}i \\ 0 & \sqrt{2}i & 0 \end{pmatrix}, \tag{21}
$$

where the first three terms are the bare qutrit eigenstates and the last matrix is written in the old basis $(\left|0\right\rangle, \left|1\right\rangle, \left|2\right\rangle)$. The outer degrees of freedom $(1,3)$ is truncated to the lowest two degrees levels (i.e. an effective qubit) as is standard, where $\left|\uparrow\right\rangle$ and $\left|\downarrow\right\rangle$ will denote the excited and ground state, respectively.

We now switch to a rotating frame corresponding to the external field, which means performing a unitary transformation with the operator:

$$
U_{\text{ext}} = U_{\text{ext},1} U_{\text{ext},2} U_{\text{ext},3}, \tag{22}
$$

where

$$
U_{\text{ext},\alpha} = \mathrm{e}^{i\omega_{\text{ext}}t/2} \left|\downarrow\middle\rangle\!\middle\langle\downarrow\right| + \mathrm{e}^{-i\omega_{\text{ext}}t/2} \left|\uparrow\middle\rangle\!\middle\langle\uparrow\right|, \tag{23}
$$

for $\alpha = 1, 3$, and

$$
U_{\text{ext},2}(t) = \mathrm{e}^{i3\omega_{\text{ext}}t/2} \left|\tilde{0}\middle\rangle\!\middle\langle\tilde{0}\right| + \mathrm{e}^{i\omega_{\text{ext}}t/2} \left|\tilde{1}\middle\rangle\!\middle\langle\tilde{1}\right| + \mathrm{e}^{-i\omega_{\text{ext}}t/2} \left|\tilde{2}\middle\rangle\!\middle\langle\tilde{2}\right|. \tag{24}
$$

We are trying to obtain a mixing of the first and second energy levels and therefore assume that we can tune ω_{ext} so that it is close to $E_2 - E_1$ and far from $E_1 - E_0$ and $E_2 - E_0$, effectively enabling us to perform the two-level approximation. A transformation of the Hamiltonian can now be performed according to the standard transformation rule

$$
H \to H^R = U_{\text{ext}}^\dagger H \, U_{\text{ext}} + i \frac{\mathrm{d}U_{\text{ext}}^\dagger}{\mathrm{d}t} U_{\text{ext}}. \tag{25}
$$

Since our Hamiltonian is quite big, we take this one part at a time. Starting with the terms purely related to the ψ_2 degree of freedom, it is a good idea to look at the matrix elements from the last factor in (21) when performing this transformation:

$$
\left\langle\tilde{1}\right| \Big(\left|1\middle\rangle\!\middle\langle 0\right| - \sqrt{2}\left|1\middle\rangle\!\middle\langle 2\right|\Big) \left|\tilde{2}\right\rangle = -\frac{\sqrt{2}(6T_2 + E_2)}{\sqrt{(6\sqrt{2}T_2)^2 + {E_2}^2}} \tag{26}
$$

$$
\left\langle\tilde{2}\right| \Big(-\left|0\middle\rangle\!\middle\langle 1\right| + \sqrt{2}\left|2\middle\rangle\!\middle\langle 1\right|\Big) \left|\tilde{1}\right\rangle = \frac{\sqrt{2}(6T_2 + E_2)}{\sqrt{(6\sqrt{2}T_2)^2 + {E_2}^2}}, \tag{27}
$$

where the rest are either irrelevant or zero. So, we get in the rotating frame

$$
\begin{aligned}
H_2 &= E_0 \left|\tilde{0}\middle\rangle\!\middle\langle\tilde{0}\right| + E_1 \left|\tilde{1}\middle\rangle\!\middle\langle\tilde{1}\right| + E_2 \left|\tilde{2}\middle\rangle\!\middle\langle\tilde{2}\right| \\
&\quad + i\frac{T_{\text{ext}}}{2} \frac{\sqrt{2}(6T_2 + E_2)}{\sqrt{(6\sqrt{2}T_2)^2 + {E_2}^2}} \left(\mathrm{e}^{i\omega_{\text{ext}}t} + \mathrm{e}^{-i\omega_{\text{ext}}t}\right) \left(-\left|\tilde{1}\middle\rangle\!\middle\langle\tilde{2}\right| \mathrm{e}^{-i\omega_{\text{ext}}t} + \left|\tilde{2}\middle\rangle\!\middle\langle\tilde{1}\right| \mathrm{e}^{i\omega_{\text{ext}}t}\right) \\
&\quad + \frac{3\omega_{\text{ext}}}{2} \left|\tilde{0}\middle\rangle\!\middle\langle\tilde{0}\right| + \frac{\omega_{\text{ext}}}{2} \left|\tilde{1}\middle\rangle\!\middle\langle\tilde{1}\right| - \frac{\omega_{\text{ext}}}{2} \left|\tilde{2}\middle\rangle\!\middle\langle\tilde{2}\right| \\
&= \Big(E_0 + \frac{3\omega_{\text{ext}}}{2}\Big) \left|\tilde{0}\middle\rangle\!\middle\langle\tilde{0}\right| + \Big(E_1 + \frac{\omega_{\text{ext}}}{2}\Big) \left|\tilde{1}\middle\rangle\!\middle\langle\tilde{1}\right| + \Big(E_2 - \frac{\omega_{\text{ext}}}{2}\Big) \left|\tilde{2}\middle\rangle\!\middle\langle\tilde{2}\right| \\
&\quad - i\Delta\Big[\left|\tilde{1}\middle\rangle\!\middle\langle\tilde{2}\right| (1 + \mathrm{e}^{-2i\omega_{\text{ext}}t}) - \left|\tilde{2}\middle\rangle\!\middle\langle\tilde{1}\right| (1 + \mathrm{e}^{2i\omega_{\text{ext}}t})\Big] \\
&\simeq \Big(E_0 + \frac{3\omega_{\text{ext}}}{2}\Big) \left|\tilde{0}\middle\rangle\!\middle\langle\tilde{0}\right| + \Big(E_1 + \frac{\omega_{\text{ext}}}{2}\Big) \left|\tilde{1}\middle\rangle\!\middle\langle\tilde{1}\right| + \Big(E_2 - \frac{\omega_{\text{ext}}}{2}\Big) \left|\tilde{2}\middle\rangle\!\middle\langle\tilde{2}\right| + i\Delta\Big(\left|\tilde{2}\middle\rangle\!\middle\langle\tilde{1}\right| - \left|\tilde{1}\middle\rangle\!\middle\langle\tilde{2}\right|\Big),
\end{aligned}
\tag{28}
$$

where we in the last equation has used the rotating wave approximation to remove the fast oscillating terms $\exp(\pm 2i\omega_{\text{ext}}t)$, and we have defined

$$\Delta = \frac{T_{\text{ext}}}{2} \frac{\sqrt{2}(E_2 + 6T_2)}{\sqrt{(6\sqrt{2}T_2)^2 + {E_2}^2}}. \tag{29}$$

Writing this in terms of the detuning from resonance

$$\delta = E_2 - E_1 - \omega_{\text{ext}}, \tag{30}$$

we can rewrite the expression above as

$$H_2^R = \left(\frac{E_1 + E_2}{2} + \xi - \frac{3\delta}{2}\right) |\tilde{0}\rangle\langle\tilde{0}| + \left(\frac{E_1 + E_2}{2} - \frac{\delta}{2}\right) |\tilde{1}\rangle\langle\tilde{1}| + \left(\frac{E_1 + E_2}{2} + \frac{\delta}{2}\right) |\tilde{2}\rangle\langle\tilde{2}| + i\Delta\Big(|\tilde{2}\rangle\langle\tilde{1}| - |\tilde{1}\rangle\langle\tilde{2}| \Big) \tag{31}$$

or, representing this in the matrix representation in the tilde states as basis states:

$$H_2^R \sim \begin{pmatrix} \xi - \frac{3\delta}{2} & 0 & 0 \\ 0 & -\frac{\delta}{2} & -i\Delta \\ 0 & i\Delta & \frac{\delta}{2} \end{pmatrix} + \frac{E_1 + E_2}{2}\mathbb{I}_3,$$

where $\mathbb{I}_3$ is the 3×3 identity matrix of the qutrit, which can be safely ignored, and $\xi = E_2 - E_1 - (E_1 - E_0) < 0$ is the absolute anharmonicity between the first and second level in the qutrit. This can be diagonalized to find the energy spectrum

$$\begin{aligned} E_0' &= \xi - \frac{3\delta}{2} \\ E_1' &= -\gamma \\ E_2' &= +\gamma, \end{aligned} \tag{32}$$

where

$$\gamma = \frac{1}{2}\sqrt{\delta^2 + 4\Delta^2}. \tag{33}$$

The (normalized) eigenstates of this Hamiltonian, expressed in the basis $\{|\tilde{0}\rangle, |\tilde{1}\rangle, |\tilde{2}\rangle\}$, are

$$\begin{aligned} |0'\rangle &\sim \begin{pmatrix} 1 \\ 0 \\ 0 \end{pmatrix} \\ |1'\rangle &\sim \frac{1}{\sqrt{\Delta^2 + \left(\frac{\delta}{2} - \gamma\right)^2}} \begin{pmatrix} 0 \\ i\Delta \\ -\frac{\delta}{2} + \gamma \end{pmatrix} \\ |2'\rangle &\sim \frac{1}{\sqrt{\Delta^2 + \left(\frac{\delta}{2} + \gamma\right)^2}} \begin{pmatrix} 0 \\ -i\Delta \\ \frac{\delta}{2} + \gamma \end{pmatrix}. \end{aligned} \tag{34}$$

In the limit $\Delta \to 0$, these reduce to the bare energy states $|i'\rangle \to |\tilde{i}\rangle$ for $i = 0, 1, 2$ when $\delta > 0$ meaning the driving frequency is below the undriven energy difference between the upper qutrit states. The Hamiltonian for the ψ_2 degree of freedom reduces to

$$H_2^R = E_0' |0'\rangle\langle 0'| + E_1' |1'\rangle\langle 1'| + E_2' |2'\rangle\langle 2'| = E_0'\mathbb{I}_2 + (E_1' - E_0') |1'\rangle\langle 1'| + (E_2' - E_0') |2'\rangle\langle 2'|, \tag{35}$$

where the diagonal term will again be throw away from this point onwards. In conclusion, we see that by tuning A_{ext} and/or ω_{ext}, we can change E_1' and E_2', i.e. the contribution of the part of the Hamiltonian purely related to ψ_2 to the energy of the two highest qutrit states.

Next, we perform the transformation to the rotating picture to the parts of the Hamiltonian purely related to the outer fluxes $j = 1, 3$. We choose the qubit spin-up state as the excited state, e.g. we associate $b_j^\dagger b_j \mapsto \frac{1}{2} + \frac{1}{2}\sigma_j^z$. Thus

$$H_j \to H_j^R = U_{\mathrm{ext},j}^\dagger \left[\sqrt{\frac{4\alpha_j}{C_j}} \left(b_j^\dagger b_j + \frac{1}{2} \right) - \frac{\beta_j}{4\alpha_j C_j} \left(b_j^\dagger + b_j \right)^4 \right] U_{\mathrm{ext},\alpha} \tag{36}$$

$$= \frac{1}{2} \left(\sqrt{\frac{4\alpha_j}{C_j}} - \frac{3\beta_j}{\alpha_j C_j} \right) \sigma_j^z + \frac{\omega_{\mathrm{ext}}}{2} \left|\downarrow\right\rangle\!\left\langle\downarrow\right| - \frac{\omega_{\mathrm{ext}}}{2} \left|\uparrow\right\rangle\!\left\langle\uparrow\right| \tag{37}$$

$$= \frac{1}{2} \left(\sqrt{\frac{4\alpha_j}{C_j}} - \frac{3\beta_j}{\alpha_j C_j} - \omega_{\mathrm{ext}} \right) \sigma_j^z, \tag{38}$$

where we have made the usual truncation to a qubit.

We have yet to do the transformation to the rotating picture for the interaction terms. The factors involved are $(b_j^\dagger + b_j)$, which normally for a qubit maps to σ_j^x before moving to the interaction picture, and $(b_j^\dagger + b_j)^2$, which maps to $2 + \sigma_j^z$ for a qubit. We start by looking at the factor (for $j = 1, 3$):

$$(b_2^\dagger + b_2)(b_j^\dagger + b_j) \to U_{\mathrm{ext},2}^\dagger \left(b_2^\dagger + b_2 \right) U_{\mathrm{ext},2} \; U_{\mathrm{ext},j}^\dagger \left(b_j^\dagger + b_j \right) U_{\mathrm{ext},j}.$$

The first factor is:

$$U_{\mathrm{ext},2}^\dagger \left(b_2^\dagger + b_2 \right) U_{\mathrm{ext},2} = k_0 \left(\mathrm{e}^{-i\omega_{\mathrm{ext}}t} \left|\tilde{0}\right\rangle\!\left\langle\tilde{1}\right| + \mathrm{e}^{i\omega_{\mathrm{ext}}t} \left|\tilde{1}\right\rangle\!\left\langle\tilde{0}\right| \right) + k_2 \left(\mathrm{e}^{-i\omega_{\mathrm{ext}}t} \left|\tilde{1}\right\rangle\!\left\langle\tilde{2}\right| + \mathrm{e}^{i\omega_{\mathrm{ext}}t} \left|\tilde{2}\right\rangle\!\left\langle\tilde{1}\right| \right), \tag{39}$$

where

$$k_i = (-1)^{\frac{i}{2}} \frac{\sqrt{2}(6T_2 - E_i)}{\sqrt{(6\sqrt{2}T_2)^2 + {E_i}^2}} \tag{40}$$

for $i = 0, 2$. The second factor contributes with:

$$U_{\mathrm{ext},j}^\dagger (b_j^\dagger + b_j) U_{\mathrm{ext},j} = \mathrm{e}^{-i\omega_{\mathrm{ext}}t} \left|\downarrow\right\rangle\!\left\langle\uparrow\right| + \mathrm{e}^{i\omega_{\mathrm{ext}}t} \left|\uparrow\right\rangle\!\left\langle\downarrow\right|. \tag{41}$$

So:

$$(b_2^\dagger + b_2)(b_j^\dagger + b_j) \to k_0 \left(\sigma_j^+ \left|\tilde{0}\right\rangle\!\left\langle\tilde{1}\right| + \sigma_j^- \left|\tilde{1}\right\rangle\!\left\langle\tilde{0}\right| + \mathrm{e}^{-i2\omega_{\mathrm{ext}}t} \sigma_j^- \left|\tilde{0}\right\rangle\!\left\langle\tilde{1}\right| + \mathrm{e}^{i2\omega_{\mathrm{ext}}t} \sigma_j^+ \left|\tilde{1}\right\rangle\!\left\langle\tilde{0}\right| \right) \tag{42}$$

$$+ k_2 \left(\sigma_j^+ \left|\tilde{1}\right\rangle\!\left\langle\tilde{2}\right| + \sigma_j^- \left|\tilde{2}\right\rangle\!\left\langle\tilde{1}\right| + \mathrm{e}^{-i2\omega_{\mathrm{ext}}t} \sigma_j^- \left|\tilde{1}\right\rangle\!\left\langle\tilde{2}\right| + \mathrm{e}^{i2\omega_{\mathrm{ext}}t} \sigma_j^+ \left|\tilde{2}\right\rangle\!\left\langle\tilde{1}\right| \right) \tag{43}$$

$$\simeq k_0 \left(\sigma_j^+ \left|\tilde{0}\right\rangle\!\left\langle\tilde{1}\right| + \sigma_j^- \left|\tilde{1}\right\rangle\!\left\langle\tilde{0}\right| \right) + k_2 \left(\sigma_j^+ \left|\tilde{1}\right\rangle\!\left\langle\tilde{2}\right| + \sigma_j^- \left|\tilde{2}\right\rangle\!\left\langle\tilde{1}\right| \right), \tag{44}$$

where we in the last equality have used the rotating wave approximation to remove the fast oscillating terms. Since we want to look at the primed mixed states as the new tunable qutrit, we transform this to the primed basis:

$$(b_2^\dagger + b_2)(b_j^\dagger + b_j) \to k_0 \sigma_j^+ \left\langle \tilde{1} | 1' \right\rangle \left|0'\right\rangle\!\left\langle 1'\right| + k_0 \sigma_j^- \left\langle 1' | \tilde{1} \right\rangle \left|1'\right\rangle\!\left\langle 0'\right| \tag{45}$$

$$+ k_2 \Big(\sigma_j^+ \left\langle 1' | \tilde{1} \right\rangle \left\langle \tilde{2} | 1' \right\rangle + \sigma_j^- \left\langle 1' | \tilde{2} \right\rangle \left\langle \tilde{1} | 1' \right\rangle \Big) \left|1'\right\rangle\!\left\langle 1'\right| \tag{46}$$

$$+ k_2 \Big(\sigma_j^+ \left\langle 1' | \tilde{1} \right\rangle \left\langle \tilde{2} | 2' \right\rangle + \sigma_j^- \left\langle 1' | \tilde{2} \right\rangle \left\langle \tilde{1} | 2' \right\rangle \Big) \left|1'\right\rangle\!\left\langle 2'\right| \tag{47}$$

$$+ k_2 \Big(\sigma_j^+ \left\langle 2' | \tilde{1} \right\rangle \left\langle \tilde{2} | 1' \right\rangle + \sigma_j^- \left\langle 2' | \tilde{2} \right\rangle \left\langle \tilde{1} | 1' \right\rangle \Big) \left|2'\right\rangle\!\left\langle 1'\right| \tag{48}$$

$$+ k_2 \Big(\sigma_j^+ \left\langle 2' | \tilde{1} \right\rangle \left\langle \tilde{2} | 2' \right\rangle + \sigma_j^- \left\langle 2' | \tilde{2} \right\rangle \left\langle \tilde{1} | 2' \right\rangle \Big) \left|2'\right\rangle\!\left\langle 2'\right| \tag{49}$$

$$+ k_0 \sigma_j^+ \left\langle \tilde{1} | 2' \right\rangle \left|0'\right\rangle\!\left\langle 2'\right| + k_0 \sigma_j^- \left\langle 2' | \tilde{1} \right\rangle \left|2'\right\rangle\!\left\langle 0'\right|. \tag{50}$$

Some of these terms look troubling, but luckily, all terms proportional with an off-diagonal overlap between the primed and tilde states will be much smaller than the diagonal ones and can be ignored. Also, all these terms are general energy non-conserving and thus could also be eliminated using a rotating wave approximation in the interaction picture. We note

that the imaginary factor from the matrix elements can be eliminated by defining $|1'\rangle \mapsto |1'\rangle_{\text{new}} = -i\,|1'\rangle_{\text{old}}$. We thus end with:

$$(b_2^\dagger + b_2)(b_j^\dagger + b_j) \xrightarrow{\text{R}} \frac{k_0\Delta}{\sqrt{\Delta^2 + \left(\frac{\delta}{2} - \gamma\right)^2}} \left(\sigma_j^+ \,|0'\rangle\langle 1'| + \sigma_j^- \,|1'\rangle\langle 0'|\right) \tag{51}$$

$$+ \frac{k_2\left(\frac{\delta}{2} + \gamma\right)}{2\gamma} \left(\sigma_j^+ \,|1'\rangle\langle 2'| + \sigma_j^- \,|2'\rangle\langle 1'|\right), \tag{52}$$

where the "R" denotes the transformation to the rotating frame. We note that the couplings $|0'\rangle \leftrightarrow |1'\rangle$ and $|1'\rangle \leftrightarrow |2'\rangle$ are not equal. In fact, for small Δ, the latter is a factor of $\sqrt{2}$ bigger, originating from the definition of the bosonic step operators.

We can now look at the transformation of the last kind of interaction term:

$$\left(b_2^\dagger + b_2\right)^2 \left(b_j^\dagger + b_j\right)^2 \to U_{\text{ext},2}^\dagger \left(b_2^\dagger + b_2\right)^2 U_{\text{ext},2}\; U_{\text{ext},j}^\dagger \left(b_j^\dagger + b_j\right)^2 U_{\text{ext},j}. \tag{53}$$

For the second factor, the transformation is equal to the identity for the standard qubit basis, i.e.

$$U_{\text{ext},j}^\dagger (b_j^\dagger + b_j)^2 U_{\text{ext},j} \mapsto 2 + \sigma_j^z. \tag{54}$$

Performing the transformation for the first factor, we get:

$$U_{\text{ext},2}^\dagger \left(b_2^\dagger + b_2\right)^2 U_{\text{ext},2} = C_{00}\,|\tilde{0}\rangle\langle\tilde{0}| + 3\,|\tilde{1}\rangle\langle\tilde{1}| + C_{22}\,|\tilde{2}\rangle\langle\tilde{2}|$$
$$+ C_{02}\Big(\,\mathrm{e}^{-i2\omega_{\text{ext}}t}\,|\tilde{0}\rangle\langle\tilde{2}| + \,\mathrm{e}^{i2\omega_{\text{ext}}t}\,|\tilde{2}\rangle\langle\tilde{0}|\,\Big). \tag{55}$$

Again, the last two terms are fast-rotating and can be removed. We have defined:

$$\begin{aligned}
C_{00} &= 1 - \frac{4E_0\left(6T_2 - E_0\right)}{\left(6\sqrt{2}T_2\right)^2 + {E_0}^2} \\
C_{02} &= \frac{4E_0E_2 - 12T_2(E_0 + E_2)}{\sqrt{\left(6\sqrt{2}T_2\right)^2 + {E_0}^2}\sqrt{\left(6\sqrt{2}T_2\right)^2 {E_2}^2}} \\
C_{22} &= 1 - \frac{4E_2\left(6T_2 - E_2\right)}{\left(6\sqrt{2}T_2\right)^2 + {E_2}^2}.
\end{aligned} \tag{56}$$

Moving to the primed basis, we find:

$$\begin{aligned}
\left(b_2^\dagger + b_2\right)^2 \to\; & C_{00}\,|0'\rangle\langle 0'| + \Big(3\big|\langle 1'|\tilde{1}\rangle\big|^2 + C_{22}\big|\langle 1'|\tilde{2}\rangle\big|^2\Big)\,|1'\rangle\langle 1'| \\
& + \Big(3\,\langle 1'|\tilde{1}\rangle\,\langle\tilde{1}|2'\rangle + C_{22}\,\langle 1'|\tilde{2}\rangle\,\langle\tilde{2}|2'\rangle\,\Big)\,|1'\rangle\langle 2'| + \Big(3\,\langle 2'|\tilde{1}\rangle\,\langle\tilde{1}|1'\rangle + C_{22}\,\langle 2'|\tilde{2}\rangle\,\langle\tilde{2}|1'\rangle\,\Big)\,|2'\rangle\langle 1'| \\
& + \Big(3\big|\langle 2'|\tilde{1}\rangle\big|^2 + C_{22}\big|\langle 2'|\tilde{2}\rangle\big|^2\Big)\,|2'\rangle\langle 2'|\,.
\end{aligned}$$

Again, we will ignore the energy non-conserving terms proportional to an off-diagonal overlap. We will, though, keep the terms proportional to a non-diagonal overlap in the energy conserving terms for accuracy of the final Hamiltonian. Evaluating the matrix elements gives:

$$\begin{aligned}
\left(b_2^\dagger + b_2\right)^2 \xrightarrow{\text{R}}\; & C_{00}\,|0'\rangle\langle 0'| + \frac{3\Delta^2 + C_{22}\left(-\frac{\delta}{2} + \gamma\right)^2}{\Delta^2 + \left(-\frac{\delta}{2} + \gamma\right)^2}\,|1'\rangle\langle 1'| \\
& + \frac{3\Delta^2 + C_{22}\left(\frac{\delta}{2} + \gamma\right)^2}{\Delta^2 + \left(\frac{\delta}{2} + \gamma\right)^2}\,|2'\rangle\langle 2'|\,.
\end{aligned} \tag{57}$$

We are now ready to look at the full transformed Hamiltonian.

Full Hamiltonian

We can now write down the full Hamiltonian for the system when coupled to an external field mixing the first and second excited state in the rotating frame of the Hamiltonian $H_{\text{ext}} = -\frac{3\omega_{\text{ext}}}{2}|\tilde{0}\rangle\langle\tilde{0}| - \frac{\omega_{\text{ext}}}{2}|\tilde{1}\rangle\langle\tilde{1}| + \frac{\omega_{\text{ext}}}{2}|\tilde{2}\rangle\langle\tilde{2}|$. We start from the Hamiltonian in equation (9) and now insert how the factors containing the bosonic step operators transform under such a transformation, as calculated in the previous section. To sum, up, we found:

$$
\begin{aligned}
T_1 b_2^\dagger b_2 - T_2\left(b_2^\dagger + b_2\right)^4 + iT_{\text{ext}}\cos(\omega_{\text{ext}}t)\left(b_2^\dagger - b_2\right) &\mapsto (E_1' - E_0')|1'\rangle\langle 1'| + (E_2' - E_0')|2'\rangle\langle 2'| \\
\sqrt{\frac{4\alpha_j}{C_j}}\left(b_j^\dagger b_j + \frac{1}{2}\right) - \frac{\beta_j}{4\alpha_j C_j}\left(b_j^\dagger + b_j\right)^4 &\mapsto \frac{1}{2}\left(\sqrt{\frac{4\alpha_j}{C_j}} - \frac{3\beta_j}{\alpha_j C_j} - \omega_{\text{ext}}\right)\sigma_j^z \\
(b_2^\dagger + b_2)(b_j^\dagger + b_j) &\mapsto \frac{k_0\Delta}{\sqrt{\Delta^2 + \left(\frac{\delta}{2} - \gamma\right)^2}}\left(\sigma_j^+|0'\rangle\langle 1'| + \sigma_j^-|1'\rangle\langle 0'|\right) \\
&\quad + \frac{k_2\left(\frac{\delta}{2} + \gamma\right)}{2\gamma}\left(\sigma_j^+|1'\rangle\langle 2'| + \sigma_j^-|2'\rangle\langle 1'|\right) \\
\left(b_2^\dagger + b_2\right)^2\left(b_j^\dagger + b_j\right)^2 &\mapsto \left(C_{00}\mathbb{I}_2 + D_1|1'\rangle\langle 1'| + D_2|2'\rangle\langle 2'|\right)\left(2 + \sigma_j^z\right),
\end{aligned}
\tag{58}
$$

where

$$
\begin{aligned}
D_1 &= \frac{3\Delta^2 + C_{22}\left(-\frac{\delta}{2} + \gamma\right)^2}{\Delta^2 + \left(-\frac{\delta}{2} + \gamma\right)^2} - C_{00} \\
D_2 &= \frac{3\Delta^2 + C_{22}\left(\frac{\delta}{2} + \gamma\right)^2}{\Delta^2 + \left(\frac{\delta}{2} + \gamma\right)^2} - C_{00}.
\end{aligned}
\tag{59}
$$

We remind the reader that we have defined the excited state as $|\uparrow\rangle$ and the ground state as $|\downarrow\rangle$, which explains the sign in front of the σ^zs. We can now write out the full Hamiltonian in the rotating frame, changing the indices from $1, 2, 3$ to L, M, R for the sake of visualizing the system as a chain of two qubits with a qutrit in the middle:

$$
\begin{aligned}
H_{\text{full}} = &\frac{\Delta_L}{2}\sigma_L^z + \Delta_M|1'\rangle\langle 1'| + (\Delta_M + \delta_M)|2'\rangle\langle 2'| + \frac{\Delta_R}{2}\sigma_R^z \\
&+ J_{LM_{01}}\left(\sigma_L^-|1'\rangle\langle 0'| + \sigma_L^+|0'\rangle\langle 1'|\right) + J_{RM_{01}}\left(\sigma_R^-|1'\rangle\langle 0'| + \sigma_R^+|0'\rangle\langle 1'|\right) \\
&+ J_{LM_{12}}\left(\sigma_L^-|2'\rangle\langle 1'| + \sigma_L^+|1'\rangle\langle 2'|\right) + J_{RM_{12}}\left(\sigma_R^-|2'\rangle\langle 1'| + \sigma_R^+|1'\rangle\langle 2'|\right) \\
&+ J_{LM}^{(z)}\sigma_L^z\left(D_1|1'\rangle\langle 1'| + D_2|2'\rangle\langle 2'|\right) + J_{RM}^{(z)}\sigma_R^z\left(D_1|1'\rangle\langle 1'| + D_2|2'\rangle\langle 2'|\right),
\end{aligned}
\tag{60}
$$

Here, the diagonal constants are

$$
\begin{aligned}
\Delta_L &= -\frac{3\beta_1}{\alpha_1 C_1} + \sqrt{\frac{4\alpha_1}{C_1}} - \frac{C_{00}}{64}\frac{E_{J1}\cos\left(\frac{\Phi_{\Sigma 1}}{2}\right)}{\sqrt{\alpha_1\alpha_2 C_1(C_1 + C_2 + C_{\text{ext}})}} - \omega_{\text{ext}} \\
\Delta_M &= E_1' - E_0' - \frac{D_1}{64}\frac{E_{J1}\cos\left(\frac{\Phi_{\Sigma 1}}{2}\right)}{\sqrt{\alpha_1\alpha_2 C_1(C_1 + C_2 + C_{\text{ext}})}} - \frac{D_1}{64}\frac{E_{J2}\cos\left(\frac{\Phi_{\Sigma 2}}{2}\right)}{\sqrt{\alpha_3\alpha_2 C_2(C_1 + C_2 + C_{\text{ext}})}} \\
\delta_M &= 2\gamma - \frac{D_2 - D_1}{64}\frac{E_{J1}\cos\left(\frac{\Phi_{\Sigma 1}}{2}\right)}{\sqrt{\alpha_1\alpha_2 C_1(C_1 + C_2 + C_{\text{ext}})}} - \frac{D_2 - D_1}{64}\frac{E_{J2}\cos\left(\frac{\Phi_{\Sigma 2}}{2}\right)}{\sqrt{\alpha_3\alpha_2 C_2(C_1 + C_2 + C_{\text{ext}})}} \\
\Delta_R &= -\frac{3\beta_3}{\alpha_3 C_2} + \sqrt{\frac{4\alpha_3}{C_2}} - \frac{C_{00}}{64}\frac{E_{J2}\cos\left(\frac{\Phi_{\Sigma 2}}{2}\right)}{\sqrt{\alpha_3\alpha_2 C_2(C_1 + C_2 + C_{\text{ext}})}} - \omega_{\text{ext}},
\end{aligned}
\tag{61}
$$

while the D_i constants are defined in (59) and the rest are

$$
\begin{aligned}
J_{LM_{01}} &= \left(\frac{1}{8L_1} - \frac{1}{8\tilde{L}_1}\right) \frac{k_0}{2(\alpha_1\alpha_2 C_1(C_1+C_2+C_{\text{ext}}))^{\frac{1}{4}}} \frac{\Delta}{\sqrt{\Delta^2 + \left(-\frac{\delta}{2}+\gamma\right)^2}} \\
J_{RM_{01}} &= \left(\frac{1}{8L_2} - \frac{1}{8\tilde{L}_2}\right) \frac{k_0}{2(\alpha_3\alpha_2 C_2(C_1+C_2+C_{\text{ext}}))^{\frac{1}{4}}} \frac{\Delta}{\sqrt{\Delta^2 + \left(-\frac{\delta}{2}+\gamma\right)^2}} \\
J_{LM_{12}} &= \left(\frac{1}{8L_1} - \frac{1}{8\tilde{L}_1}\right) \frac{k_2}{4(\alpha_1\alpha_2 C_1(C_1+C_2+C_{\text{ext}}))^{\frac{1}{4}}} \frac{\frac{\delta}{2}+\gamma}{\gamma} \\
J_{RM_{12}} &= \left(\frac{1}{8L_2} - \frac{1}{8\tilde{L}_2}\right) \frac{k_2}{4(\alpha_3\alpha_2 C_2(C_1+C_2+C_{\text{ext}}))^{\frac{1}{4}}} \frac{\frac{\delta}{2}+\gamma}{\gamma} \\
J_{LM}^{(z)} &= -\frac{E_{J1}}{128} \frac{\cos\left(\frac{\Phi_{\Sigma 1}}{2}\right)}{\sqrt{\alpha_1\alpha_2 C_1(C_1+C_2+C_{\text{ext}})}} \\
J_{RM}^{(z)} &= -\frac{E_{J2}}{128} \frac{\cos\left(\frac{\Phi_{\Sigma 2}}{2}\right)}{\sqrt{\alpha_3\alpha_2 C_2(C_1+C_2+C_{\text{ext}})}}.
\end{aligned} \tag{62}
$$

To sum up, we have now, starting from the circuit in Supplementary Fig. 1, calculated the resulting Hamiltonian and added a dynamical tuning of the qutrit via driving of the first and second excited bare qutrit energy levels. In doing so, we first transformed to the rotating picture with respect to the driving field and then to the new mixed qutrit eigenstates, finally obtaining the Hamiltonian above.

Supplementary Note 2: Circuit parameters used in the simulations

In Table 0.1, we have included a list of realistic circuit parameters and the corresponding spin-model parameters they yield. We can split this into three parts, a circuit with Hamiltonian parameters suited to implementing the dissociation procedure, a circuit implementing the ACSWAP gate, and a circuit with all levels off-resonant, suitable to implementing the STIRAP procedure and CCZ and holonomic gates. Note that for the dissociation and ACSWAP procedures, we are working in the frame rotating with the AC Stark drive frequency, ω_{ext}, and thus the energy terms are reduced accordingly. This of course does not change the (non-trivial) dynamics, since only the relative energy differences are important.

Supplementary Note 3: Direct Dissociation to Entanglement

We wish to obtain an entangled state between the state where qubit one and three is in the excited state and the state where they are both in the ground state. Since the Hamiltonian conserves the total projection of spin, we start in the state $|\downarrow 2 \downarrow\rangle$. We look at the matrix representation of our Hamiltonian in the basis $\{\, |\downarrow 2 \downarrow\rangle, |\downarrow 1 \uparrow\rangle, |\uparrow 1 \downarrow\rangle, |\uparrow 0 \uparrow\rangle \,\}$. If we assume the system to be symmetric, i.e. we do not distinguish between $\alpha = L$ and $\alpha = R$, and further assume the states to be resonant, i.e. $\Delta_M = \Delta_R = \Delta_L = \delta_M$, the contribution from these energy terms is proportional to the identity and we are left with the matrix representation of the XX and ZZ-terms, which we can easily diagonalize to find the eigenvalues. In the simplified case $J_{\alpha M_{01}} = J_{\alpha M_{12}} = J_{\alpha M}$ and $D_1 = D_2 = 1$ (This is not essential, and is only chosen as to ease the readability of the analysis. The equation for $D_2 J_{\alpha M}^{(z)}$ in the main text is the result in the non-simplified case), the eigenvalues are:

$$
E_1 = 0 \quad , \quad E_2 = -2J_{\alpha M}^{(z)} \quad , \quad E_3 = -J_{\alpha M}^{(z)} - \lambda \quad \text{and} \quad E_4 = -J_{\alpha M}^{(z)} + \lambda, \tag{63}
$$

where

$$
\lambda = \sqrt{4{J_{\alpha M}}^2 + {J_{\alpha M}^{(z)}}^2}. \tag{64}
$$

	Circuit parameters	Spin-model parameters
Dissociation, first part (STIRAP)	$L_{1,2} = 19.999\,\mathrm{nH}$,<br>$\tilde{L}_{1,2} = 18.713\,\mathrm{nH}$,<br>$E_{J1,2}/(2\pi) = 185.91$ GHz,<br>$E_{Jq1,3}/(2\pi) = 79.515$ GHz,<br>$E_{Jq2}/(2\pi) = 27.441$ GHz,<br>$C_{1,2} = 55.816$ fF,<br>$\Phi_{\Sigma 1,2} = -0.43452\,\Phi_0$,<br>$C_{\mathrm{ext}} = 2.3411$ fF,<br>Dynamical driving off	$\Delta_L/(2\pi) = 15.271$ GHz,<br>$\Delta_M/(2\pi) = 13.841$ GHz,<br>$\delta_M/(2\pi) = 13.671$ GHz,<br>$\Delta_R/(2\pi) = 15.271$ GHz,<br>$J_{\alpha M_{01}}/(2\pi) = 9.2737$ MHz,<br>$J_{\alpha M_{12}}/(2\pi) = 12.996$ MHz,<br>$J_{\alpha M}^{(z)}/(2\pi) = -20.433$ MHz,<br>$D_1 = 1.9877$,<br>$D_2 = 3.9754$,<br>$\max(\Omega_{1,2})/(2\pi) = 20$ MHz
Dissociation, second part (two-photon resonance)	$L_{1,2} = 20.000\,\mathrm{nH}$,<br>$\tilde{L}_{1,2} = 16.949\,\mathrm{nH}$,<br>$E_{J1,2}/(2\pi) = 32.371$ GHz,<br>$E_{Jq1,3}/(2\pi) = 109.74$ GHz,<br>$E_{Jq2}/(2\pi) = 108.94$ GHz,<br>$C_{1,2} = 55.935$ fF,<br>$\Phi_{\Sigma 1,2} = 0.27790\,\Phi_0$,<br>$C_{\mathrm{ext}} = 71.773$ fF,<br>$A_{\mathrm{ext}}/(4\alpha_2(C_1+C_2+C_{\mathrm{ext}}))^{-1/4} = 0.1000$,<br>$\omega_{\mathrm{ext}}/(2\pi) = 14.674$ GHz	In the frame rotating with ω_{ext}:<br>$\Delta_L/(2\pi) = 0.46529$ GHz,<br>$\Delta_M/(2\pi) = 0.088376$ GHz,<br>$\delta_M/(2\pi) = 0.84222$ GHz,<br>$\Delta_R/(2\pi) = 0.46529$ GHz,<br>$J_{\alpha M_{01}}/(2\pi) = 15.0203$ MHz,<br>$J_{\alpha M_{12}}/(2\pi) = 17.114$ MHz,<br>$J_{\alpha M}^{(z)}/(2\pi) = -6.4890$ MHz,<br>$D_1 = 2.6636$,<br>$D_2 = 3.3015$
CCNOT/CCZ	Same as during STIRAP.	Same as for STIRAP, except<br>$\Omega_1/(2\pi) = 6$ MHz
ACSWAP<br>(first part of CCSWAP)	$L_{1,2} = 16.049\,\mathrm{nH}$,<br>$\tilde{L}_{1,2} = 18.988\,\mathrm{nH}$,<br>$E_{J1,2}/(2\pi) = 56.08$ GHz,<br>$E_{Jq1,3}/(2\pi) = 155.22$ GHz,<br>$E_{Jq2}/(2\pi) = 135.33$ GHz,<br>$C_{1,2} = 56.619$ fF,<br>$\Phi_{\Sigma 1,2} = 0.49999\,\Phi_0$,<br>$C_{\mathrm{ext}} = 90.011$ fF,<br>$A_{\mathrm{ext}}/(4\alpha_2(C_1+C_2+C_{\mathrm{ext}}))^{-1/4} = 0.10000$,<br>$\omega_{\mathrm{ext}}/(2\pi) = 14.367$ GHz	In the frame rotating with ω_{ext}:<br>$\Delta_L/(2\pi) = 0.9113$ GHz,<br>$\Delta_M/(2\pi) = -0.0798$ GHz,<br>$\delta_M/(2\pi) = 0.9121$ GHz,<br>$\Delta_R/(2\pi) = 0.9113$ GHz,<br>$J_{\alpha M_{01}}/(2\pi) = 14.311$ MHz,<br>$J_{\alpha M_{12}}/(2\pi) = 15.173$ MHz,<br>$J_{\alpha M}^{(z)}/(2\pi) = -0.601$ kHz,<br>$D_1 = 2.8377$,<br>$D_2 = 3.12544$
Holonomic gate (double-controlled)	Same as during STIRAP	Same as for STIRAP, except<br>$\Omega_{1,2}/(2\pi) = 15$ MHz

Table 0.1: A table of realistic parameters and the corresponding Hamiltonian parameters used in each implementation. In all simulations, we have included finite relaxation and coherence times set to $T_1 = 31\,\mu\mathrm{s}$ and $T_2 = 35\,\mu\mathrm{s}$, respectively. The parameters of the second part of the CSWAP is not shown, but is obtained by varying only the external parameters relative to the first part. The resulting parameters are similar to what is shown for the CCNOT gate implementation.

The associated eigenvectors are

$$|v_1\rangle = \frac{1}{\sqrt{2}}\begin{pmatrix} 0 \\ 0 \\ -1 \\ 1 \end{pmatrix} \quad , \quad |v_2\rangle = \frac{1}{\sqrt{2}}\begin{pmatrix} -1 \\ 1 \\ 0 \\ 0 \end{pmatrix} \quad , \quad |v_3\rangle = \sqrt{\frac{\lambda - J_{\alpha M}^{(z)}}{4\lambda}}\begin{pmatrix} \frac{2J_{\alpha M}}{J_{\alpha M}^{(z)} - \lambda} \\ \frac{2J_{\alpha M}}{J_{\alpha M}^{(z)} - \lambda} \\ 1 \\ 1 \end{pmatrix}$$

$$\text{and} \quad |v_4\rangle = \sqrt{\frac{\lambda + J_{\alpha M}^{(z)}}{4\lambda}}\begin{pmatrix} \frac{2J_{\alpha M}}{J_{\alpha M}^{(z)} + \lambda} \\ \frac{2J_{\alpha M}}{J_{\alpha M}^{(z)} + \lambda} \\ 1 \\ 1 \end{pmatrix}. \qquad (65)$$

We can now find

$$\left\langle f\middle|\mathrm{e}^{-iHt}\middle|\downarrow 2\downarrow\right\rangle = \sum_{n,m=1}^{4} \left\langle f|v_n\right\rangle \left\langle v_n\middle|\mathrm{e}^{-iHt}\middle|v_m\right\rangle \left\langle v_m|\downarrow 2\downarrow\right\rangle$$
$$= \sum_{n=1}^{4} \left\langle f|v_n\right\rangle \mathrm{e}^{-iE_n t} \left\langle v_n|\downarrow 2\downarrow\right\rangle, \tag{66}$$

where $|f\rangle$ is one of the four basis states mentioned earlier. From this, the complete time development of $|\downarrow 2\downarrow\rangle$ is recovered analytically. Specifically, we have

$$\left|\left\langle\downarrow 2\downarrow\middle|\mathrm{e}^{-iHt}\middle|\downarrow 2\downarrow\right\rangle\right|^2 = \frac{1}{4}\Big(\cos\Big(J^{(z)}_{\alpha M}t\Big) + \cos(\lambda t)\Big)^2 + \frac{1}{4}\Big(\sin\Big(J^{(z)}_{\alpha M}t\Big) + \frac{J^{(z)}_{\alpha M}}{\lambda}\sin(\lambda t)\Big)^2$$

and

$$\left|\left\langle\uparrow 0\uparrow\middle|\mathrm{e}^{-iHt}\middle|\downarrow 2\downarrow\right\rangle\right|^2 = \frac{1}{4}\Big(\cos\Big(J^{(z)}_{\alpha M}t\Big) - \cos(\lambda t)\Big)^2 + \frac{1}{4}\Big(\sin\Big(J^{(z)}_{\alpha M}t\Big) - \frac{J^{(z)}_{\alpha M}}{\lambda}\sin(\lambda t)\Big)^2.$$

Because $\lambda = \sqrt{4{J_{\alpha M}}^2 + {J^{(z)}_{\alpha M}}^2}$ we, for a general $J^{(z)}_{\alpha M}$, expect the probabilities to have a chaotic behavior. We ask ourselves if there exists a certain value of $J^{(z)}_{\alpha M} > 0$ where we can find a time t at which

$$\left|\left\langle\downarrow 2\downarrow\middle|\mathrm{e}^{-iHt}\middle|\downarrow 2\downarrow\right\rangle\right|^2 = \left|\left\langle\uparrow 0\uparrow\middle|\mathrm{e}^{-iHt}\middle|\downarrow 2\downarrow\right\rangle\right|^2 = \frac{1}{2} \tag{67}$$

and

$$\frac{\mathrm{d}}{\mathrm{d}t}\left|\left\langle\downarrow 2\downarrow\middle|\mathrm{e}^{-iHt}\middle|\downarrow 2\downarrow\right\rangle\right|^2 = \frac{\mathrm{d}}{\mathrm{d}t}\left|\left\langle\uparrow 0\uparrow\middle|\mathrm{e}^{-iHt}\middle|\downarrow 2\downarrow\right\rangle\right|^2 = 0. \tag{68}$$

From the first equality in (67), we find the condition

$$\cos(\lambda t)\cos\Big(J^{(z)}_{\alpha M}t\Big) = -\frac{J^{(z)}_{\alpha M}}{\lambda}\sin(\lambda t)\sin\Big(J^{(z)}_{\alpha M}t\Big), \tag{69}$$

while we from equation (68) get

$$\sin(\lambda t)\,\Big(\cos\Big(J^{(z)}_{\alpha M}t\Big) + \cos(\lambda t)\Big) = 0 = \sin(\lambda t)\,\Big(\cos\Big(J^{(z)}_{\alpha M}t\Big) - \cos(\lambda t)\Big)$$
$$\Rightarrow \quad \sin(\lambda t) = 0 \qquad \text{or} \qquad \cos\Big(J^{(z)}_{\alpha M}t\Big) = \cos(\lambda t) = 0. \tag{70}$$

Here, the second option implies that $\sin\Big(J^{(z)}_{\alpha M}t\Big) = \pm\sin(\lambda t) = \pm 1 \neq 0$, which is not compatible with the condition (69). Therefore, we must have $\sin(\lambda t) = 0$ and from (69) also $\cos\Big(J^{(z)}_{\alpha M}t\Big) = 0$. This means that we have two conditions on our time $t > 0$:

$$t = n\frac{\pi}{\lambda} \quad \text{and} \quad t = m\frac{\pi}{2J^{(z)}_{\alpha M}}\ ,\ n \in \mathbb{N},\ m = 1, 3, 5, \ldots \tag{71}$$

We solve this for $J^{(z)}_{\alpha M}$:

$$n\frac{\pi}{\lambda} = m\frac{\pi}{2J^{(z)}_{\alpha M}} \Rightarrow 4n^2{J^{(z)}_{\alpha M}}^2 = (4{J_{\alpha M}}^2 + {J^{(z)}_{\alpha M}}^2)m^2$$
$$\Rightarrow J^{(z)}_{\alpha M} = \frac{2J_{\alpha M}}{\sqrt{4n^2/m^2 - 1}}, \tag{72}$$

where $n = 1, 2, 3, \ldots$ and $m = 1, 3, 5, \ldots$ with the further condition to keep the time real $4n^2/m^2 > 1 \Rightarrow n > m/2$. This is plotted for $n = m = 1$ in Supplementary Fig. 2 for arbitrary values of parameters for proof of concept.

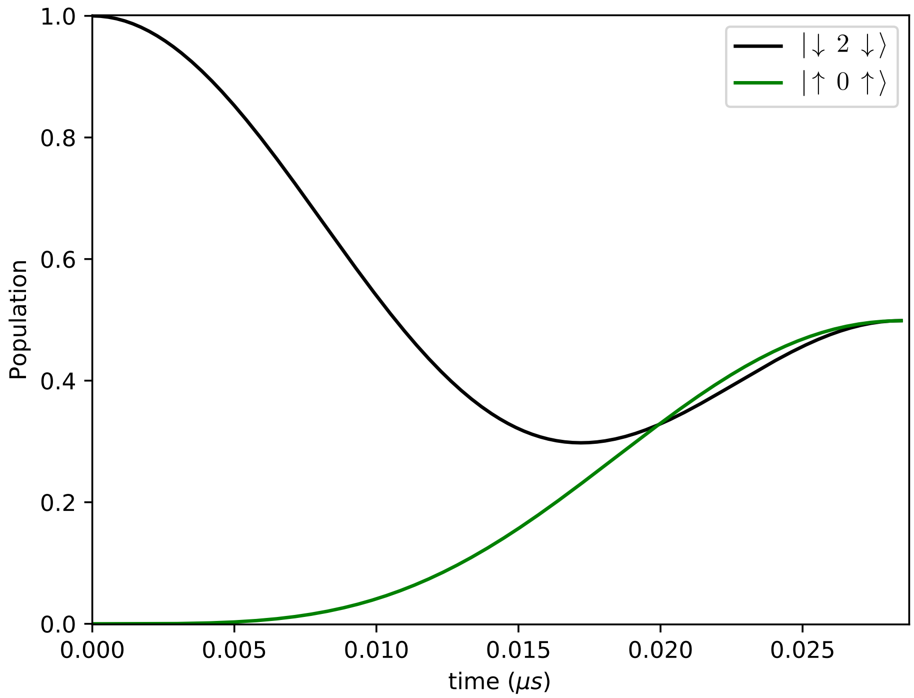

Supplementary Figure 2: Probability of occupation in the case $J^{(z)}_{\alpha M} = 2J_{\alpha M}/\sqrt{3}$. We notice that the desired entangled state is reached at $t = \pi/\lambda = \pi/2J^{(z)}_{\alpha M} \approx 1.36\frac{1}{J_{\alpha M}} \approx 0.028\,\mu\text{s}$.

Supplementary Note 4: Analysis of the CSWAP

Assuming that the excited states of the outer qubits are in resonance with the second excited state of the qutrit ($\Delta_L \simeq \delta_M \simeq \Delta_R$) while the qutrit ground state is far detuned from the rest, we can move to the interaction picture of the diagonal in equation (60), ignoring these terms and also all terms involving the ground state ($|0\rangle$) of the qutrit. We also choose to assume symmetry between both ends of the chain, so e.g. $J_{LM_{12}} = J_{RM_{12}} = J_{\alpha M_{12}}$, and that $J^{(z)}_{\alpha M} = 0$. A non-zero ZZ-coupling will make the states with the qubits in the same state slightly detuned, introducing a non-perfect transition. This can be remedied a bit by choosing $\Delta_\alpha = \delta_M - 2\frac{D_1+D_2}{2}J^{(z)}_{\alpha M}$, but because in general $D_1 \neq D_2$, this detuning can not be fixed completely. In the basis $\{|\downarrow 1 \downarrow\rangle, |\uparrow 1 \downarrow\rangle, |\downarrow 2 \downarrow\rangle, |\downarrow 1 \uparrow\rangle, |\uparrow 2 \downarrow\rangle, |\uparrow 1 \uparrow\rangle, |\downarrow 2 \uparrow\rangle, |\uparrow 2 \uparrow\rangle\}$, the interaction part of the Hamiltonian can be written very simply:

$$H_I = \left(\begin{array}{c|ccc|ccc|c} 0 & 0 & 0 & 0 & 0 & 0 & 0 & 0 \\ \hline 0 & 0 & J_{\alpha M_{12}} & 0 & 0 & 0 & 0 & 0 \\ 0 & J_{\alpha M_{12}} & 0 & J_{\alpha M_{12}} & 0 & 0 & 0 & 0 \\ 0 & 0 & J_{\alpha M_{12}} & 0 & 0 & 0 & 0 & 0 \\ \hline 0 & 0 & 0 & 0 & 0 & J_{\alpha M_{12}} & 0 & 0 \\ 0 & 0 & 0 & 0 & J_{\alpha M_{12}} & 0 & J_{\alpha M_{12}} & 0 \\ 0 & 0 & 0 & 0 & 0 & J_{\alpha M_{12}} & 0 & 0 \\ \hline 0 & 0 & 0 & 0 & 0 & 0 & 0 & 0 \end{array}\right). \tag{73}$$

Notice the block matrix form where the two 3×3 matrices represent irreducible subspaces. Furthermore, they are equal in form, so when performing the time evolution $\mathrm{e}^{-iH_I t}$ we only have to exponentiate one of these irreducible sub-matrices. It is easily found that:

$$\exp\left[-i\begin{pmatrix} 0 & J_{\alpha M_{12}} & 0 \\ J_{\alpha M_{12}} & 0 & J_{\alpha M_{12}} \\ 0 & J_{\alpha M_{12}} & 0 \end{pmatrix} t\right] = \frac{1}{2}\begin{pmatrix} \cos\left(\sqrt{2}J_{\alpha M_{12}}t\right) + 1 & -i\sqrt{2}\sin\left(\sqrt{2}J_{\alpha M_{12}}t\right) & \cos\left(\sqrt{2}J_{\alpha M_{12}}t\right) - 1 \\ -i\sqrt{2}\sin\left(\sqrt{2}J_{\alpha M_{12}}t\right) & 2\cos\left(\sqrt{2}J_{\alpha M_{12}}t\right) & -i\sqrt{2}\sin\left(\sqrt{2}J_{\alpha M_{12}}t\right) \\ \cos\left(\sqrt{2}J_{\alpha M_{12}}t\right) - 1 & -i\sqrt{2}\sin\left(\sqrt{2}J_{\alpha M_{12}}t\right) & \cos\left(\sqrt{2}J_{\alpha M_{12}}t\right) + 1 \end{pmatrix}. \tag{74}$$

Letting the qutrit start in the ground state, we can now find

$$\mathrm{e}^{-iH_I t} \left|\downarrow 1 \downarrow\right\rangle = \left|\downarrow 1 \downarrow\right\rangle \tag{75}$$

$$\mathrm{e}^{-iH_I t} \left|\uparrow 1 \downarrow\right\rangle = \cos^2\left(\frac{J_{\alpha M_{12}}}{\sqrt{2}} t\right) \left|\uparrow 1 \downarrow\right\rangle - \frac{i}{\sqrt{2}} \sin\left(\sqrt{2} J_{\alpha M_{12}} t\right) \left|\downarrow 2 \downarrow\right\rangle - \sin^2\left(\frac{J_{\alpha M_{12}}}{\sqrt{2}} t\right) \left|\downarrow 1 \uparrow\right\rangle \tag{76}$$

$$\mathrm{e}^{-iH_I t} \left|\uparrow 1 \uparrow\right\rangle = -\frac{i}{\sqrt{2}} \sin\left(\sqrt{2} J_{\alpha M_{12}} t\right) \left(\left|\uparrow 2 \downarrow\right\rangle + \left|\downarrow 2 \uparrow\right\rangle \right) + \cos\left(\sqrt{2} J_{\alpha M_{12}} t\right) \left|\uparrow 1 \uparrow\right\rangle . \tag{77}$$

We wish to obtain a -CSWAP, so we require $\left| \left\langle \downarrow 1 \uparrow \right| \mathrm{e}^{-iH_I t} \left|\uparrow 1 \downarrow\right\rangle \right|^2 = 1$, thus we must choose the operation time to be $T = \frac{\pi}{\sqrt{2} J_{\alpha M_{12}}}$. Inserting this into the equations above, we get

$$\left\langle \downarrow 1 \uparrow \right| \mathrm{e}^{-iH_I T} \left|\uparrow 1 \downarrow\right\rangle = \left\langle \uparrow 1 \uparrow \right| \mathrm{e}^{-iH_I T} \left|\uparrow 1 \uparrow\right\rangle = -1 = -\left\langle \downarrow 1 \downarrow \right| \mathrm{e}^{-iH_I T} \left|\downarrow 1 \downarrow\right\rangle . \tag{78}$$

This last matrix element has the wrong sign, so if we tried to perform a -CSWAP between the outer qubits in different superpositions of the 'up' and 'down' state, we would obtain an unfortunate relative sign change. This can be seen by assuming the left qubit starts in the superposition

$$\left|\psi_{\mathrm{L}}\right\rangle = a \left|\uparrow\right\rangle + b \left|\downarrow\right\rangle , \tag{79}$$

while the right qubit starts in the superposition

$$\left|\psi_{\mathrm{R}}\right\rangle = c \left|\uparrow\right\rangle + d \left|\downarrow\right\rangle . \tag{80}$$

Here, a, b, c and d are complex coefficients. The total starting state of the system is then the factorizable state

$$\left(a \left|\uparrow\right\rangle + b \left|\downarrow\right\rangle\right) \left|1\right\rangle \left(c \left|\uparrow\right\rangle + d \left|\downarrow\right\rangle\right) = ac \left|\uparrow 1 \uparrow\right\rangle + ad \left|\uparrow 1 \downarrow\right\rangle + bc \left|\downarrow 1 \uparrow\right\rangle + bd \left|\downarrow 1 \downarrow\right\rangle . \tag{81}$$

Operating on this state with the unitary operator $\mathrm{e}^{-iH_I T}$ now gives:

$$-ac \left|\uparrow 1 \uparrow\right\rangle - ad \left|\downarrow 1 \uparrow\right\rangle - bc \left|\uparrow 1 \downarrow\right\rangle + bd \left|\downarrow 1 \downarrow\right\rangle . \tag{82}$$

This state is not factorizable because of the sign difference between the first term and the rest, and therefore does not have a simple interpretation as the system where a SWAP operation has been performed.

This can be fixed by first operating with a CCZ gate on the system so that only this specific state ($\left|\downarrow 1 \downarrow\right\rangle$) obtains a sign change. This of course requires that the states are first moved out of resonance, but this is already required in order to catch the system in the swapped state. We then obtain the state

$$-ac \left|\uparrow 1 \uparrow\right\rangle - ad \left|\downarrow 1 \uparrow\right\rangle - bc \left|\uparrow 1 \downarrow\right\rangle - bd \left|\downarrow 1 \downarrow\right\rangle = -\left(c \left|\uparrow\right\rangle + d \left|\downarrow\right\rangle\right) \left|1\right\rangle \left(a \left|\uparrow\right\rangle + b \left|\downarrow\right\rangle\right) . \tag{83}$$

It is clear that the states of the outer qubits have been swapped, just as we wanted!

We thus end up with a CSWAP gate in the interaction picture of H_0 with a two-step operation scheme and with the control "bit" being comprised of the states $|0\rangle$ and $|1\rangle$ of the qutrit (or, equivalently, the states $|1\rangle$ and $|2\rangle$ if we instead apply the CCZ so only the state $\left|\uparrow 1 \uparrow\right\rangle$ receives a sign change). By symmetry, the topmost excited qutrit state $|2\rangle$ will also allow transfer, but here the state $\left|\uparrow 2 \uparrow\right\rangle$ will need a sign change.

A simulation of the CSWAP/Fredkin gate is shown in the main text, where we get a fidelity of around 0.95 for a perfect SWAP operation when the qutrit starts in the first excited state, as predicted by the analytical investigation above.
